# Targeting Anti-Angiogenic VEGF_165_b–VEGFR1 Signaling Promotes Nitric Oxide Independent Therapeutic Angiogenesis in Preclinical Peripheral Artery Disease Models

**DOI:** 10.3390/cells11172676

**Published:** 2022-08-28

**Authors:** Sivaraman Kuppuswamy, Brian H. Annex, Vijay C. Ganta

**Affiliations:** Vascular Biology Center and Department of Medicine, Augusta University, Augusta, GA 30912, USA

**Keywords:** angiogenesis, anti-angiogenic VEGF-A, ischemia, growth factor signaling, nitric oxide, diabetes

## Abstract

Nitric oxide (NO) is the critical regulator of VEGFR2-induced angiogenesis. Neither VEGF-A over-expression nor L-Arginine (NO-precursor) supplementation has been effective in helping patients with Peripheral Artery Disease (PAD) in clinical trials. One incompletely studied reason may be due to the presence of the less characterized anti-angiogenic VEGF-A (VEGF_165_b) isoform. We have recently shown that VEGF_165_b inhibits ischemic angiogenesis by blocking VEGFR1, not VEGFR2 activation. Here we wanted to determine whether VEGF_165_b inhibition using a monoclonal isoform-specific antibody against VEGF_165_b vs. control, improved perfusion recovery in preclinical PAD models that have impaired VEGFR2-NO signaling, including (1) type-2 diabetic model, (2) endothelial Nitric oxide synthase-knock out mice, and (3) Myoglobin transgenic mice that have impaired NO bioavailability. In all PAD models, VEGF_165_b inhibition vs. control enhanced perfusion recovery, increased microvascular density in the ischemic limb, and activated VEGFR1-STAT3 signaling. In vitro, VEGF_165_b inhibition vs. control enhanced a VEGFR1-dependent endothelial survival/proliferation and angiogenic capacity. These data demonstrate that VEGF_165_b inhibition induces VEGFR1-STAT3 activation, which does not require increased NO to induce therapeutic angiogenesis in PAD. These results may have implications for advancing therapies for patients with PAD where the VEGFR2-eNOS-NO pathway is impaired.

## 1. Introduction

Atherosclerotic occlusions that develop in the lower extremities can result in a moderate to severe decrease in blood flow, resulting in tissue ischemia, which causes Peripheral Artery Disease (PAD). PAD is a large contributor to the global cardiovascular disease burden with >200 million patients worldwide [1]. Approximately ~10% of PAD patients develop a severe form of PAD, critical limb ischemia (CLI), where tissue necrosis is a common incidence and is associated with high mortality [2]. Improving blood flow by inducing angiogenesis in the ischemic muscle is an established approach to increase perfusion and improve walking and/or salvage the limb from amputation in PAD patients [3]. Angiogenesis, the formation of new blood vessels from existing vasculature by vascular endothelial growth factor (VEGF)-A is perhaps the most extensively studied angiogenic pathway.

One of the major mechanisms by which VEGF-A regulates angiogenesis is by activating VEGFR2-AKT-ERK-eNOS signaling, which produces NO to induce angiogenesis. However, despite its ability to induce a strong pro-angiogenic phenotype in vitro and in vivo PAD models, none of the VEGF-A clinical trials or NO supplementation by L-arginine showed any clinical benefit in PAD [4,5,6,7]. A possible reason for their failures in PAD clinical trials could be explained based on the relatively recent recognition of increased anti-angiogenic VEGF_165_b isoform production in ischemic muscle over pro-angiogenic VEGF_165_a isoform [8,9]. The anti-angiogenic VEGF_165_b isoforms occur by alternative splicing in the 8th exon of VEGF-A. These isoforms are considered to be ‘inactive competitive inhibitors’ of VEGFR2 signaling that act by competing with pro-angiogenic VEGF-A isoforms for binding sites on VEGFR2 [10]. Previous studies have indicated that, unlike VEGF_165_a, VEGF_165_b is a weak agonist of VEGFR2 that can induce VEGFR2-phosphorylation, but not downstream signaling, needed to elicit an angiogenic response. As such VEGF_165_b was thought to be an ‘inactive blocker’ of VEGF-A-induced VEGFR2 signaling [11,12,13]. Limited information exists on VEGF_165_b–VEGFR1 interactions in regulating endothelial function in cardiovascular pathologies [14]. We have recently shown that VEGF_165_b inhibits ischemic angiogenesis by decreasing VEGFR1 activation [8]. Additionally, a previous study by Glass et al. [14] showed the role of VEGF_165_b–VEGFR1 interactions in regulating microvascular hydraulic conductivity in rat mesenteric vessels.

The rationale of this study is based on our data that (1) in human PAD muscle biopsies increased VEGF_165_b binding to VEGFR2 correlated with increased VEGFR2 activation, whereas increased VEGF_165_b binding to VEGFR1 correlated with decreased VEGFR1 activation vs. non-PAD controls, and (2) in in vitro competition studies, exogenous VEGF_165_b-induced VEGFR2 activation almost to the same extent as VEGF_165_a in ECs and (3) VEGF_165_b inhibition-induced VEGFR1 activation without changes in VEGFR2 activation [8]. Despite the ability to induce VEGFR2 activation, exogenous VEGF_165_b treatment did not increase the angiogenic potential of normal or ischemic endothelial cells [8]. Whereas depleting VEGF_165_b using an isoform-specific monoclonal antibody against VEGF_165_b isoform-induced angiogenesis by activating VEGFR1 signaling [8,15]. Consistent with our data, previous studies by Kawamura et al. [13] and Catena et al. [12] also showed that VEGF_165_b induces VEGFR2 activation in pulmonary arterial endothelial (PAE) cells and Chinese Hamster Ovarian (CHO) cells that express VEGFR2, respectively [12,13]. On the contrary, Li et al. [16] have shown that overexpressing VEGF_165_b in ovarian cancer cell lines, including SKOV3 and OVCAR3, inhibits VEGFR2 signaling. However, the role of VEGF_165_b in regulating VEGFR1 vs. VEGFR2 signaling in ischemic endothelial cells is not clear.

Based on these data, we questioned whether VEGFR1 activation achieved by VEGF_165_b inhibition has the ability to induce ischemic angiogenesis even when VEGFR2-eNOS-NO signaling is impaired e.g., diabetic-PAD [17,18]. We used three preclinical PAD models, including (1) C57BL/6 mice on a high-fat diet (type 2 diabetic model well known to have impaired VEGFR2-eNOS-NO signaling [17,18], (2) eNOS deficient mice (eNOS-KO, that have defective NO generation [19]), and (3) Myoglobin transgenic mice (Mg-Tg, which show defective angiogenesis due to impaired Nitric Oxide (NO) bioavailability [20,21]) in ischemic muscle to determine whether VEGFR1 activation is sufficient to induce angiogenesis and increase perfusion recovery in PAD.

## 2. Materials and Methods

### 2.1. Cells and Cell Culture

Human Umbilical Vein endothelial cells (HUVECs) were purchased and cultured in an all-in-one complete endothelial growth medium that contains 5 mM Glucose (Cat No.: 211-500) from Cell Applications Inc. (San Diego, CA, USA). HUVECs were purchased from Cell Applications and cultured in HUVEC growth medium [8]. C57BL/6 Mouse Primary Skeletal Muscle Microvascular Endothelial Cells (Cat No.: C57-6220) were purchased and cultured in Complete Mouse Endothelial Cell Medium (Cat: M1168) from Cell Biologics (Chicago, IL, USA).

### 2.2. Recombinant Proteins and Reagents

Recombinant human VEGF_165_a (Cat No.: 293-VE) and recombinant human/murine VEGF_165_b (Cat No.: 3045-VE) were purchased from R&D (Minneapolis, MN, USA). Recombinant mouse VEGF_165_a (Cat No.: 200-34) was purchased from Shenandoah Biotechnology (Warwick, PA, USA). STATTIC (Cat No.: 27-981-0) was purchased from Tocris Biosciences (Minneapolis, MN, USA). BSA-Palmitate Saturated Fatty Acid Complex (5 mM, Cat No.: 29558) was purchased from Cayman Chemicals (Ann Arbor, MI, USA).

### 2.3. Cell Transfection

Non-targeting (Cat No.: D-001206-13-20) and VEGFR1 siRNA (Cat No.: M-003136-03-0005) were purchased from Horizon Discovery (Cambridge, UK). RNAiMax (Cat No.: 13778150) was purchased from ThermoFisher Scientific (Waltham, MA, USA). HUVECs were transfected with 150 nM control or VEGFR1 siRNA using RNAiMAX for 48 h according to the manufacturer’s instructions.

### 2.4. Antibodies

Antibodies against pVEGFR1 (Y1333-Cat No.: SAB4504006, Y1048-Cat No.: SAB4504649), pVEGFR2 (Y1175, Cat No.: SAB4504567), and VEGFR2 (Cat No.: SAB4501643) were purchased from Sigma-Aldrich, St. Louis, MO, USA; Flt1 (Cat No.: SC-316, raised against C-terminus amino acids between 1288–1338, and hence cannot bind to soluble Flt1) and VEGF-A (Cat No.: SC-7269) were purchased from Santa Cruz Biotechnology Inc., Dallas, TX, USA; VEGFR1 (Cat No.: PA5-16493) and CD31 (Cat No.: MA3105) purchased from ThermoFisher Scientific, Grand Island, NY, USA; VEGFR2 (Cat No.: 55B11), pAKT (Cat No.: 4060), AKT (Cat No.: 4691), pERK1/2 (Cat No.: 4370), ERK1/2 (Cat No.: 9102), pSTAT3 (Cat No.: 9145), and STAT3 (Cat No.: 12640) were purchased for Cell Signaling and Technology, Danvers, MA, USA; eNOS (Cat No.: 610299) was purchased from BD Pharmingen, San Jose, CA, USA; peNOS (Cat No.: 66127) from Abcam, Cambridge, MA, USA; VEGF-B (Cat No.: MAB751) and VEGF_165_b (Cat No.: MAB3045), used in Western blotting as well as to block VEGF_165_b in vitro and in vivo, was purchased from R&D, Minneapolis, MN, USA. PlGF antibody (Cat No.: SC1883, Santa Cruz Biotechnology) was a kind gift from Dr. Yao Liang Tang, Vascular Biology Center, Augusta University.

### 2.5. Cell Treatments

Confluent endothelial cells were treated with VEGF_165_a (50 ng/mL) or VEGF_165_b (50 ng/mL) for 10 min under normal growth conditions. HUVECs were challenged with hypoxia serum starvation for 24 h followed by treatment with VEGF_165_a (50 ng/mL) or VEGF_165_b (50 ng/mL) for 10 min.

### 2.6. Hypoxia Serum Starvation

Cells were incubated in an endothelial starvation medium purchased from Cell applications Inc. that contained 5 mM Glucose (Cat No.: 209-250) and were subjected to hypoxia (2% O2) for 24 h [8,15].

### 2.7. STAT3 Inhibition

Confluent ECs were treated with varying concentrations of STATTIC ranging from 0.1 μM to 1.0 μM for 24 h under normal growth conditions or HSS. Control cells received the same volume of DMSO as 1.0 μM STATTIC.

### 2.8. Palmitic Acid Treatment

Confluent ECs were treated with 500 μM BSA-Palmitate under HSS conditions for 24 h. Control cells received the same amount of BSA matching the amount of BSA-Palmitate.

### 2.9. In Vitro Diabetic-PAD Model

As an in vitro model for diabetic-PAD, HUVECs were incubated with 500 μM BSA-Palmitate in endothelial starvation medium and challenged with 2% hypoxia for 24 h. HUVECs treated with 500 μM BSA-Palmitate in a normal growth medium under normal growth conditions were used as a model for diabetes. Cells treated with an equal volume of BSA matching BSA-Palmitate treatment were used as a control in these experiments.

### 2.10. In Vitro Angiogenesis (Capillary-like Loop Formation) Assay

Endothelial cells post-treatments were trypsinized and the cell number was quantified using Corning CytoSmart cell counter. An equal number of cells (~15,000) were plated on growth factor reduced matrigel (Cat No.: 356231, R&D) to avoid any interference from VEGF-A present in the matrigel. Capillary-like tubes formed on the matrigel were photographed at 1 h, 2 h, and 3 h at the center concave, and the number of nodes was quantified by at least 2 people who were blinded to the treatments using NIH Image J Angiogenesis Analyzer [8,22].

### 2.11. Cell Proliferation

Endothelial cells were plated in 96 well plates at equal density. Post-treatment, cells were incubated with CCK9 at 1–2 h according to the manufacturer’s instructions. Post incubation, the plates were colorimetrically read at 450 nM using a BioTek Synergy LX Multi-Mode Plate Reader.

### 2.12. Mice

All animal experiments were approved by the Augusta University IACUC and conformed to the Guide for the Care and Use of Laboratory Animals published by the US National Institutes of Health. C57BL/6 mice on 60 kCal (high-fat diet) for 4 months were purchased from Jackson laboratories (DIO C57BL/6J Mice). Only males on a high-fat diet were used in T2D-PAD studies. Fasting glucose levels in these mice on a high-fat diet were measured by performing a glucose tolerance test using One Touch Ultra Glucometer and Strips according to our previous publications [23,24]. The basal glucose levels in the fasting mice used in the study were 189.7 ± 12.8 mg/dL, and the approximate weight of these mice at the time of performing hind limb ischemia experiments was ~43.6 ± 2.1 gm. Wild type C57BL/6, eNOS-KO mice, and Myoglobin transgenic mice were bred in the University of Virginia and Augusta University animal breeding facility. Both males and females were used in eNOS-KO and Mg-Tg studies.

### 2.13. Murine Model of Hind Limb Ischemia (HLI) and Microvascular Blood Flow Quantification

HLI was performed as described previously [8,16]. A combination of Ketamine (Ketathesia, (ZooPharm, 90 mg/kg) and Xylazine (Anased, Akorn, 10 mg/kg) was injected intraperitoneally (single dose) at 10 μL/gm mouse to induce anesthesia and perform unilateral femoral artery ligation and excision as a model of experimental PAD. Briefly, the femoral artery was ligated and resected from just above the inguinal ligament to its bifurcation at the origin of saphenous and popliteal arteries. Perfusion recovery was measured by quantifying microvascular blood flow by laser Doppler imaging (Perimed, Inc., Ardmore, PA, USA) on days 0, 3, 7, and 14 post-HLI. Perfusion in the ischemic limb was normalized to a non-ischemic limb for each mouse [8,15]. Mice were sacrificed with an overdose (20 μL/gm mouse) of Ketamine and Xylazine combination followed by cervical dislocation.

### 2.14. Necrosis Scores

The extent of necrosis was scored as follows: Grade I—involving only toes, Grade II—extending to dorsum pedis, Grade III—extending to crus, and Grade IV—extending to the thigh or complete necrosis [8,15].

### 2.15. Immunostaining

Gastrocnemius muscle tissues were fixed in 4% paraformaldehyde overnight at 4 °C, followed by RT for another 24 h. Later, tissues were incubated in 30% sucrose for ~24 h (until the tissues sink to the bottom) and embedded in OCT for cryosectioning. Thick cryosections of 5 µM were processed for immunostaining 12–14. Briefly, sections were washed in PBS twice followed by antigen retrieval in citrate buffer (Cat no: H3300, Vector Laboratories, Burlingame, CA, USA), blocked in 5% goat serum for 1 h, followed by incubation in CD31 antibody overnight at 4 °C. Later, sections were washed (PBS) and incubated in secondary antibodies conjugated to AlexaFluor-555 (Cat No.: A31572, ThermoFisher Scientific, Grand Island, NY, USA) for 2 h at RT. Sections were washed (PBS) and mounted using Prolong gold anti-fade mounting media (Cat No.: P36935, ThermoFisher Scientific, Grand Island, NY, USA). Immunofluorescent images were obtained using an EVOS-M5000 fluorescent microscope.

### 2.16. Terminal Deoxy-Uridine Nick End Labeling (TUNEL) Analysis

TUNEL assay was performed using Click-iT^®^ TUNEL Alexa Fluor^®^ 488 Imaging Assay (Cat No.: C10245) purchased from ThermoFisher Scientific, Grand Island, NY, USA, according to the manufacturer’s instructions, followed by immunostaining with CD31 antibody as described above. Four random images per section were photographed on EVOS M5000 fluorescent microscope and quantified as an average of TUNEL+ ECs per image between treatment groups [8,15].

### 2.17. Western Blot Analysis

At least 50 μgm of tissue or 20 μgm of cell lysates were resolved on SDS-PAGE and transferred onto nitrocellulose membrane for Western blotting as previously described, using the iBright Imaging System [8,15,22].

### 2.18. Immunoprecipitation

~1 µg of the antibody was incubated in 50 μgm of protein lysates overnight at 4 °C in an end-to-end mixer. Antibody–antigen complexes were isolated by adding Goat-Anti-Rabbit (Cat No.: 21356) or Goat-Anti-Mouse (Cat No.: 21354) conjugated magnetic beads (ThermoFisher, Grand Island, NY, USA) for 2–3 h at room temperature. Antibody–antigen complexes were separated by boiling in sample buffer (Cat No.: BP-111R, Boston Bioproducts, Ashland, MA, USA) for 5–10 min, resolved in SDS-PAGE and Western blotted, as previously described [8,15].

### 2.19. Statistical Analysis

GraphPad Prism 9 was used to analyze the statistical significance of the data and generate graphical representations. An unpaired *t*-test was used to compare 2 specific groups. One-way ANOVA with Bonferroni select pair comparison or Dunnett’s post-tests were used to compare more than 2 groups. Repeated measures ANOVA was used for time-course laser doppler perfusion recovery measurements. A nonparametric Mann–Whitney test was used for necrosis scores. Statistical tests used to determine the significance of a specific experiment accompanied each figure legend. *p* < 0.05 is considered significant for all experiments. Data are presented as Mean ± SEM for all experiments.

## 3. Results

**VEGF_165_b activates VEGFR2 signaling in normal and hypoxia serum starved ECs in vitro.** Hypoxia is a known inducer of VEGF-A [25,26,27]. To determine whether hypoxia serum starvation (HSS), an in vitro PAD model [8,15,28,29,30] also induces VEGF-A levels, we performed a Western blot analysis of VEGF-A in normal and HSS HUVECs using a pan-VEGF-A antibody that recognizes all the VEGF-A isoforms (pro- and anti-angiogenic) [8,15,31,32,33,34]. Western blot analysis showed a numerical increase in total VEGF-A levels in HSS-challenged HUVECs vs. normal (Supplementary Figure S1). Using an isoform-specific monoclonal antibody [8], we have previously shown that HSS induces significantly higher VEGF_165_b levels in HUVECs [8]. More importantly, in human PAD, we observed that the fraction of VEGF_165_b is ~3X higher than the pro-angiogenic VEGF-A levels [8]. Taken together, our current data indicate that the fraction of pro-angiogenic VEGF_165_a is decreased, and the fraction of anti-angiogenic VEGF_165_b isoform is increased in HSS HUVECs vs. normoxic controls.

**Figure 1:**
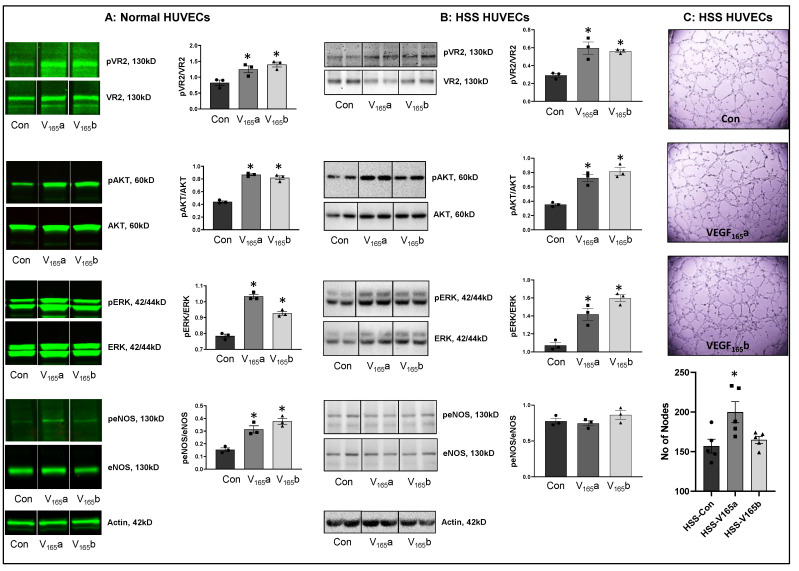
VEGF_165_b activates VEGFR2 signaling in normal and HSS-HUVECs: Immunoblot analysis of pVR2(Y1175)/VR2, pAKT/AKT, pERK/ERK, peNOS/eNOS in (**A**) HUVECs treated with VEGF_165_a (V_165_a, 50 ng/mL) or VEGF_165_b (V_165_b, 50 ng/mL) for 15 min under normal growth conditions, (**B**) HUVECs challenged with HSS for 24 h followed by treatment with V_165_a (50 ng/mL), V_165_b (50 ng/mL) or a combination of V_165_a (50 ng/mL) + V_165_b (50 ng/mL) for 15 min. *n* = 3. One-way ANOVA with Dunnett’s post-test. * *p* < 0.05 considered significant. (**C**) In vitro tube formation assay of HSS HUVECs treated with 50 ng/mL of V_165_a or V_165_b for 24 h. *n* = 5. One-way ANOVA with Dunnett’s post-test. * *p* < 0.05 considered significant. Data Mean ± SEM.

We next determined whether VEGF_165_b-induced VEGFR2-phosphorylation results in the activation of downstream AKT-ERK-eNOS signaling similar to VEGF_165_a in confluent ECs under normoxia or challenged with HSS (an in vitro model of PAD, HSS-HUVECs). The immunoblots of normal HUVECs treated with recombinant-VEGF_165_b or recombinant-VEGF_165_a showed a significant increase in pVEGFR2_Y1175_ (V_165_a: ~1.5-fold, V_165_b: ~1.7-fold), pAKT (V_165_a: ~1.9-fold, V_165_b: ~1.9-fold), pERK (V_165_a: ~1.3-fold, V_165_b: ~1.2-fold), and peNOS (V_165_a: ~2.0-fold, V_165_b: ~2.4-fold) activation almost to the same extent vs. untreated controls (Figure 1A). These data indicate that VEGF_165_b activates this VEGFR2-AKT-ERK signaling to the same extent as VEGF_165_a in normal ECs, confirming the data from previously published data from other groups [12,13,33,35].

We next examined the role of VEGF_165_b in regulating VEGFR2 signaling in HSS-HUVECs. Immunoblot analysis showed that both VEGF_165_b and VEGF_165_a induced pVEGFR2_Y1175_ (~2-fold), pAKT (~2-fold), and pERK activation to the same extent in HSS-HUVECs vs. untreated controls (Figure 1B). Interestingly, no significant difference in eNOS activation was observed in HSS-HUVECs treated with VEGF_165_a or VEGF_165_b (Figure 1B). These data indicate that VEGF_165_b is not a competitive inhibitor but an activator of VEGFR2 signaling even in HSS-HUVECs. Despite the ability of VEGF_165_b to activate the pro-angiogenic VEGFR2 signaling, VEGF_165_b did not induce the angiogenic capacity of HSS-HUVECs vs. untreated controls (Figure 1C), suggesting that the anti-angiogenic function of VEGF_165_b is not dependent on inhibiting VEGFR2 signaling. Furthermore, immunoblot analysis showed that while VEGF_165_a significantly decreased total VEGFR2 and AKT levels, VEGF_165_b treatment did not induce significant differences in their expression. Both VEGF_165_a and VEGF_165_b significantly induced ERK levels without affecting the expression of eNOS or VEGFR1 vs. untreated controls (Supplementary Figure S2).

**Figure 2:**
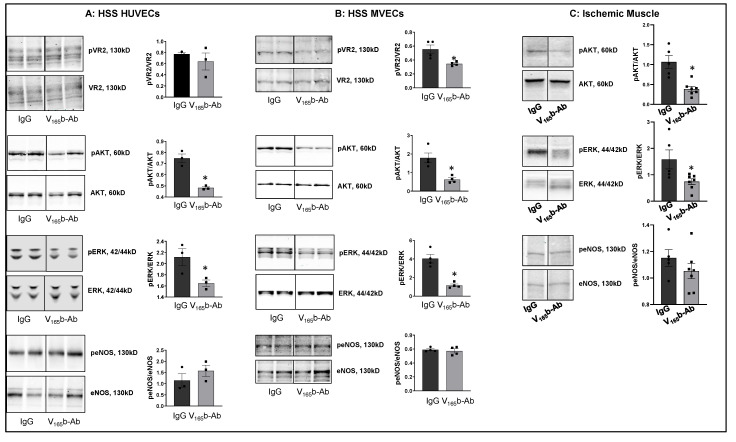
VEGF_165_b inhibition decreases VEGFR2 signaling in vitro and in vivo: Immunoblot analysis of pVR2(Y1175)/VR2, pAKT/AKT, pERK/ERK, peNOS/eNOS in (**A**) HSS-HUVECs treated with IgG or VEGF_165_b-Ab for 24 h. *n* = 3. Unpaired *t*-test. (**B**) HSS-MVECs were treated with IgG or VEGF_165_b-Ab for 24 h. *n* = 4, Unpaired *t*-test. (**C**) Immunoblot analysis of pAKT/AKT, pERK/ERK, peNOS/eNOS in Balb/c mice ischemic muscle treated with IgG or VEGF_165_b-Ab at day 3 post-HLI. *n* ≥ 5. Unpaired *t*-test. * *p* < 0.05 considered significant. Data Mean ± SEM.

**VEGF_165_b inhibition decreases VEGFR2 signaling in ischemic ECs in vitro and in vivo.** To determine the role of VEGF_165_b inhibition in regulating VEGFR1 vs. VEGFR2 activation, we treated HSS-HUVECs or HSS-mouse skeletal muscle ECs (HSS-MVECs) with isotype-matched IgG or VEGF_165_b-Ab (10 μg/mL [8,15]). HSS-HUVECs treated with VEGF_165_b-Ab showed no significant difference in pVEGFR2_Y1175_ activation but significantly decreased pAKT (~1.5-fold), and pERK (~1.3-fold) activation (Figure 2A). HSS-MVECs treated with VEGF_165_b-Ab showed a significant decrease in pVEGFR2 _Y1175_ (~1.8-fold), pAKT (~3.6-fold) and pERK (~3.9-fold) activation vs. IgG (Figure 2B). No significant difference in peNOS was observed in VEGF_165_b-Ab-treated HSS-HUVECs or HSS-MVECs vs. IgG (Figure 2A,B).

To confirm the role of VEGF_165_b inhibition in regulating VEGFR2 signaling in vivo, we performed immunoblot analysis of day 3 ischemic muscle samples treated with IgG or VEGF_165_b-Ab (200 µg, i.m, three non-overlapping sites in Balb/c mice gastrocnemius and tibialis anterior muscles). Immunoblot analysis showed that VEGF_165_b-Ab significantly decreased pAKT (~5.1-fold) and pERK (~2.9-fold) activation with no changes in peNOS activation vs. IgG (Figure 2C).

**Figure 3:**
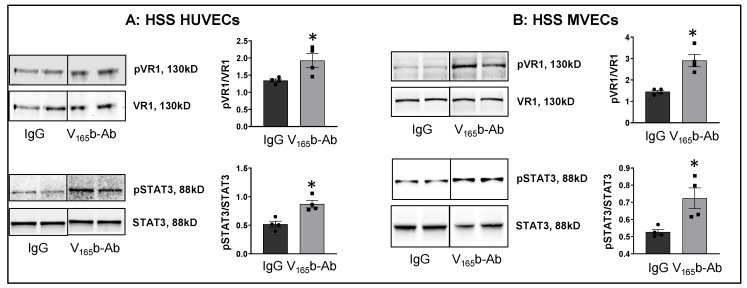
VEGF_165_b inhibition VEGFR1 activation in vitro and in vivo. Immunoblot analysis of pVR1(Y1333)/VR1 and pSTAT3/STAT3 in (**A**) HSS-HUVECs treated with IgG or VEGF_165_b-Ab for 24 h. *n* = 4. Unpaired *t*-test. (**B**) HSS-MVECs treated with IgG or VEGF_165_b-Ab for 24 h. *n* = 4. Unpaired *t*-test. * *p* < 0.05 considered significant. Data Mean ± SEM.

We next examined whether VEGF_165_b-Ab induces VEGFR1 activation in HSS-HUVECs and HSS-MVECs. Immunoblot analysis showed that VEGF_165_b-Ab-treated HSS-HUVECs and HSS-MVECs have significantly higher pVEGFR1_Y1333_ (HSS-HUVEC: ~1.6-fold, HSS-MVEC: ~1.4-fold) and pSTAT3 (HSS-HUVEC: ~1.3-fold, HSS-MVEC: ~1.6-fold) activation vs. IgG (Figure 3A,B). While HSS increases VEGF_165_b levels in HUVECs [8], a significant decrease in VEGF-B and PLGF (VEGFR1 specific ligands) levels was observed in HSS-HUVECs (Supplementary Figure S3). These data suggest that endogenously produced VEGF_165_b acts as an autocrine inhibitor of VEGFR1 in HSS-HUVECs.

**Figure 4:**
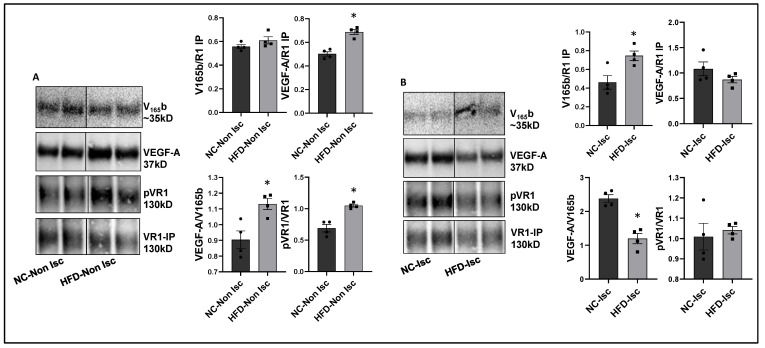
Increased binding of VEGF_165_b to VEGFR1 in HFD-ischemic muscle blocks VEGFR1 activation: (**A**) Immunoblot analysis of V_165_b, VEGF-A, and pVR1 in VEGFR1 pull-down fractions (VR1-IP (immunoprecipitated)) in normal chow- (NC) and high-fat diet (HFD)-fed C57BL/6 non-ischemic muscle. *n* = 4. Unpaired *t*-test. (**B**) Immunoblot analysis of V_165_b, VEGF-A, and pVR1 in VEGFR1 pull-down fractions (VR1-IP) in normal chow and high-fat diet-fed C57BL/6 ischemic muscle at day 3 post-HLI. *n* = 4. Unpaired *t*-test. * *p* < 0.05 considered significant. Data Mean ± SEM.

**VEGF_165_b inhibition enhances perfusion in Type-2 diabetic-PAD mice.** Immunoblot analysis to determine the relative levels of VEGF_165_b and total VEGF-A showed no significant differences between a non-ischemic high-fat diet (HFD, 60 kCal for 4 months) vs. normal chow-fed mice skeletal muscle samples (Supplementary Figure S4A). However, VEGFR1 pull-down fractions from non-ischemic HFD vs. non-ischemic normal chow samples showed a significant decrease in the fraction of VEGF_165_b bound to VEGFR1 (~1.3-fold, Figure 4A), which inversely correlated with increased pVEGFR1_Y1333_ activation in non-ischemic HFD samples vs. non-ischemic normal chow samples (~1.5-fold, Figure 4A).

We next examined the relative levels of VEGF_165_b and total VEGF-A in ischemic HFD samples vs. ischemic normal chow samples at day 3 post-HLI, a time point at which the extent of perfusion recovery is comparable between the two groups. Immunoblot analysis showed no significant difference in VEGF_165_b or total VEGF-A levels between ischemic HFD samples vs. ischemic normal chow samples (Supplementary Figure S4B). Interestingly, VEGFR1 pull-down fractions showed a significant increase in the bound VEGF_165_b fraction in HFD-ischemic samples vs. ischemic normal chow samples (~1.2-fold). No significant difference in pVEGFR1_Y1333_ activation was observed between HFD-ischemic samples vs. normal chow ischemic samples (Figure 4B) suggesting that increased VEGF_165_b binding to VEGFR1 in HFD-ischemic muscle blocked VEGFR1 activation. No significant difference in pVEGFR1_Y1333_ (Supplementary Figure S5A), pVEGFR2_Y1175_, or pAKT activation was observed in HFD-ischemic muscle vs. normal chow ischemic muscle (Supplementary Figures S4C and S5B). However, a significant increase in pERK activation was observed in HFD-ischemic muscle vs. normal chow ischemic muscle (Supplementary Figure S5D) suggesting that a VEGFR2-independent mechanism regulates ERK activation in HFD-ischemic muscle.

**Figure 5:**
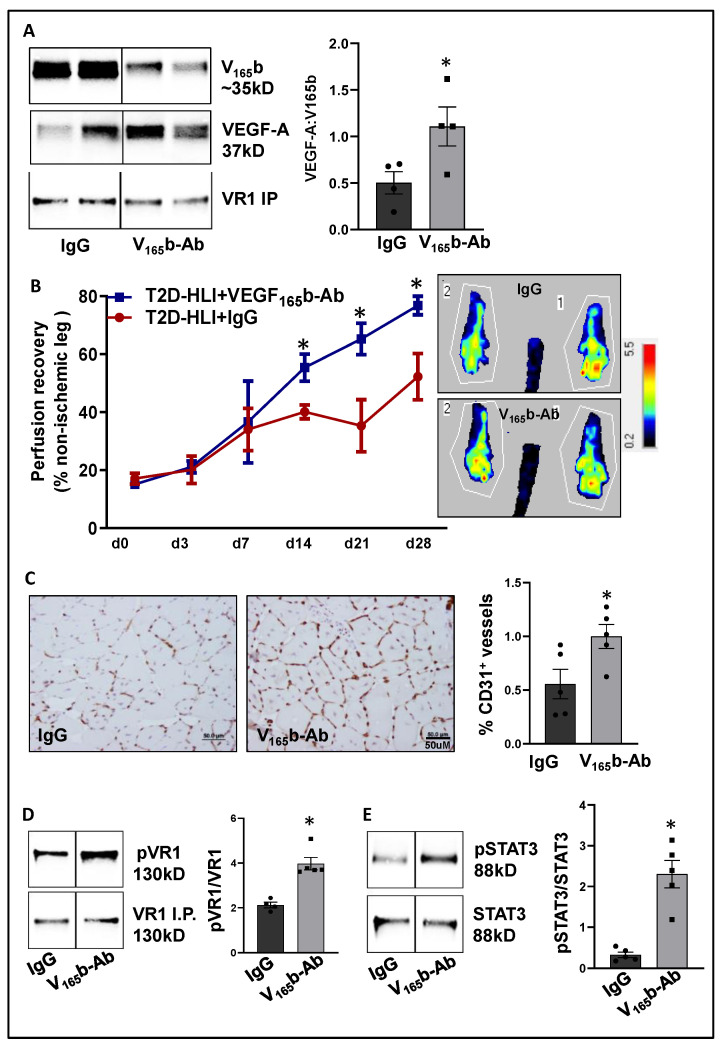
VEGF_165_b inhibition induces VEGFR1-STAT3 signaling and promotes perfusion recovery in the T2D-PAD model: (**A**) Immunoblot analysis of VEGF_165_b (V_165_b) and total VEGF-A bound to VEGFR1 in the ischemic gastrocnemius muscle of C57BL/6 mice on a HFD treated with IgG or VEGF_165_b-Ab at day 3 post-HLI. *n* = 4. Unpaired *t*-test. (**B**) Laser Doppler microvascular perfusion imaging of HFD mice treated with IgG or V_165_b over the time course of recovery. *n* = 7. Repeated Measures ANOVA with Dunnett’s post-test. * *p* < 0.05 considered significant at specific time points. (**C**) Immunohistochemical analysis of CD31 in HFD-ischemic gastrocnemius muscle treated with IgG or V_165_b-Ab at day 28 post-HLI. *n* = 7. Unpaired *t*-test. (**D**,**E**) Immunoblot analysis of pVR1(Y1333)/VR1 in VEGFR1 pull-down fractions and pSTAT3/STAT3 in HFD-ischemic gastrocnemius muscle treated with IgG or V_165_b-Ab at day 3 post-HLI. *n* = 5. Unpaired *t*-test. Significant outliers (*p* < 0.05) were excluded from the data analysis in Figure 4A by performing Grubbs’ test. Data Mean ± SEM.

After confirming that ischemia induces VEGF_165_b binding to VEGFR1 in HFD skeletal muscle, we determined the functional role of VEGF_165_b-Ab in regulating perfusion recovery in HFD-ischemic muscle. VEGF_165_b-Ab or isotype-matched IgG (200 µg/100 μL PBS/mouse) was injected intramuscularly into HFD mouse gastrocnemius (two non-overlapping sites) and tibialis anterior (one site) immediately after HLI (d0) and days 3, 7, 14, and 21 post-HLI. VEGFR1 pull-down at day 3 post HLI with IgG or VEGF_165_b-Ab-treated samples showed a significant increase in total VEGF-A bound to VEGFR1 indicating that VEGF_165_b binding to VEGFR1 was reduced by VEGF_165_b-Ab treatment (Figure 5A). Laser Doppler showed that VEGF_165_b-Ab significantly improved limb perfusion (day 14: IgG-40.1 ± 2.4 vs. V_165_b-Ab-55.35 ± 4.7; day 21: IgG-35.3 ± 9.0 vs. V_165_b-Ab-65.22 ± 5.4; day 28: IgG-52.1 ± 8 vs. V_165_b-Ab-76.7 ± 3.2) in HFD-ischemic muscle vs. IgG (Figure 5B). The immunohistochemical analysis of CD31 at day 28 post-HLI samples showed a significant increase in microvascular density in VEGF_165_b-Ab-treated HFD-ischemic muscle vs. IgG (Figure 5C). 

We next performed immunoblot analysis to determine changes in VEGFR1 vs. VEGFR2 signaling in VEGF_165_b-Ab vs. IgG-treated HFD-ischemic muscle on day 3 post-HLI. Immunoblot analysis showed a significant increase in pVEGFR1_Y1333_ (~1.5-fold, Figure 5D) and pSTAT3 (~1.5-fold, Figure 5E) activation in VEGF_165_b-Ab-treated HFD-ischemic muscle vs. IgG. While no significant differences in pVEGFR2_Y1175_, pERK, or peNOS were observed between VEGF_165_b-Ab vs. IgG-treated HFD-ischemic muscle samples, a significant increase in pAKT (~1.8-fold) activation was observed in VEGF_165_b-Ab-treated HFD-ischemic muscle vs. IgG (Supplementary Figure S6). We have previously shown that VEGFR1 directly interacts with STAT3 to induce STAT3 activation [8]. To test whether STAT3 regulates AKT activation, normal or HSS-HUVECs were treated with STAT3 inhibitor, STATTIC [36] in a dose-dependent manner. Immunoblot analysis showed that 1.0 μM STATTIC significantly decreased AKT activation in both normal and HSS-HUVECs (Supplementary Figure S7A,B), indicating that STAT3 can regulate AKT activation in the endothelium. Furthermore, the immunoblot analysis of Bcl2, Bax (downstream effectors of STAT3 in regulating apoptosis [30]), and caspase-3 at day 3 post-HLI showed a significant increase in the ratio of Bcl2/Bax (~2.9-fold, Supplementary Figure S8A) and a significant decrease in activated caspase-3 levels (~2.3-fold, Supplementary Figure S8B) in VEGF_165_b-Ab-treated HFD-ischemic muscle vs. IgG. These data suggested that activation of VEGFR1-STAT3 signaling by blocking VEGF_165_b decreased cell death and increased microvascular density to enhance perfusion recovery in HFD-ischemic muscle.

**VEGF_165_b inhibition decreases limb necrosis in eNOS-KO mice.** eNOS-KO mice have a severely impaired response to HLI. More importantly, VEGF-A superfusion failed to induce angiogenesis in eNOS-KO mice [19,37]. Hence, we used eNOS-KO mice to determine whether VEGF_165_b-Ab can improve tissue perfusion/recovery in preclinical PAD. The immunoblot analysis of VEGF_165_b and total VEGF-A at day 3 post-HLI showed a significant decrease in the ratio of VEGF-A:VEGF_165_b levels (~2.7-fold, Supplementary Figure S9A) in eNOS-KO vs. WT ischemic muscle, indicating that VEGF_165_b levels predominate in eNOS-KO mice ischemic muscle vs. WT. Furthermore, the immunoblot analysis of VEGFR1 activation showed a significant decrease in pVEGFR1 _Y1333_ activation (~1.4-fold, Supplementary Figure S9B) in eNOS-KO ischemic muscle vs. WT. These data show that a significant increase in VEGF_165_b levels decreases pVEGFR1 _Y1333_ activation in eNOS-KO ischemic muscle vs. WT. Total protein was used to normalize due to differences in Actin expression between WT vs. eNOS-KO ischemic muscle (Supplemental immunoblots for Figure 5).

**Figure 6:**
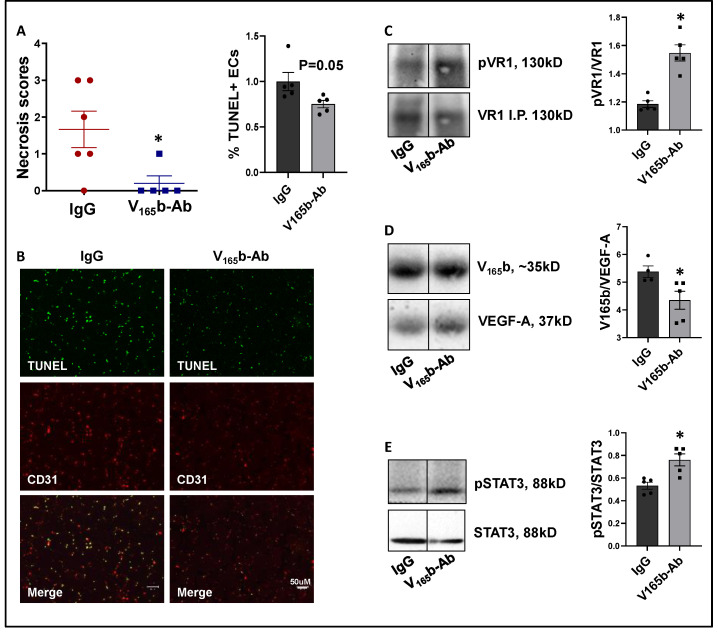
VEGF_165_b inhibition induces VEGFR1-STAT3 signaling and decreases necrosis in eNOS-KO mice in experimental PAD. (**A**) Necrosis scores in eNOS-KO mice ischemic muscle treated with IgG or VEGF_165_b-Ab. *n* ≥ 5, Non-Parametric Mann-Whitney test. (**B**) TUNEL (shown in green) and CD31 immunostaining (shown in red) to quantify endothelial apoptotic cell death in eNOS-KO mice ischemic muscle treated with IgG or V_165_b-Ab at day 3 post-HLI. *n* = 5. Unpaired *t*-test. (**C**–**E**) Immunoblot analysis of V_165_b, VEGF-A, and pVR1 in VEGFR1 pull-down fractions (VR1-IP); pSTAT3/STAT3 in eNOS-KO mice ischemic muscle treated with IgG or V_165_b-Ab at day 3 post-HLI. *n* ≥ 5. Unpaired *t*-test. * *p* < 0.05 considered significant. Data Mean ± SEM.

VEGF_165_b-Ab significantly decreased limb necrosis in eNOS-KO ischemic muscle vs. IgG-treated eNOS-KO ischemic muscle (Figure 6A). TUNEL analysis to determine the extent of EC death showed that VEGF_165_b-Ab decreased TUNEL^+^ (Green) CD31^+^ (Red) apoptotic ECs (*p* = 0.05, Figure 6B) in eNOS-KO ischemic muscle vs. IgG. We next examined whether decreased limb necrosis and cell death in VEGF_165_b-Ab-treated eNOS-KO ischemic muscle are due to activation of VEGFR1 signaling. VEGFR1 pulldown fractions in eNOS-KO ischemic muscle treated with VEGF_165_b-Ab or IgG showed a significant decrease in the fraction of VEGF_165_b bound to VEGFR1 (~1.2-fold, Figure 6C) in eNOS-KO ischemic muscle vs. IgG. A significant increase in pVEGFR1_Y1333_ activation (~1.3-fold, Figure 6D) was observed in VEGFR1 pull-down fractions in eNOS-KO ischemic muscle treated with VEGF_165_b-Ab vs. IgG. VEGFR1 activation resulted in a significant increase in pSTAT3 activation (~1.4-fold, Figure 6E) in eNOS-KO muscle treated with VEGF_165_b-Ab vs. IgG. These data indicate that decreased VEGF_165_b binding to VEGFR1 in eNOS-KO muscle treated with VEGF_165_b-Ab allowed for the activation of VEGFR1-STAT3 signaling that protected eNOS-KO ischemic muscle from necrosis.

We next performed immunoblot analysis to examine whether VEGF_165_b-Ab modulated the VEGFR2 signaling intermediates AKT and ERK. VEGF_165_b-Ab in eNOS-KO ischemic muscle did not induce any changes in pAKT activation (Supplementary Figure S10A) but induced pERK activation (~1.2-fold, Supplementary Figure S10B) vs. IgG.

**VEGF_165_b inhibition enhances perfusion in myoglobin transgenic (Mg-Tg) PAD mice that have impaired NO bioavailability**. Excessive myoglobin expression in Mg-Tg mice skeletal muscle functions as a nitric oxide sink and impairs perfusion recovery [20,21,38]. Hence, we determined whether VEGF_165_b-Ab could induce perfusion recovery despite the lack of the capacity to increase NO bioavailability. Immunoblot analysis to determine relative changes in VEGF_165_b vs. total VEGF-A at day 3 post-HLI, (a time point where the perfusion recovery between WT and Mg-Tg mice is comparable) showed no significant difference in the ratio of total VEGF-A to VEGF_165_b between WT vs. Mg-Tg ischemic muscle (Supplementary Figure S11A). Immunoblot analysis, used to determine changes in VEGFR1 activation between WT vs. Mg-Tg, showed that the total VEGFR1 levels are significantly lower in Mg-Tg ischemic muscle vs. WT (~3.5-fold decrease, (Supplementary Figure S11B). Since there was a significant decrease in total VEGFR1 levels in Mg-Tg ischemic muscle vs. WT, the extent of pVEGFR1_Y1333_ activation was compared with total protein levels (Ponceau), which showed a significant decrease in Mg-Tg ischemic muscle vs. WT, correlating with decreased VEGFR1 levels (Supplementary Figure S11B). Total protein was used to normalize due to differences in Actin expression between WT vs. Mg-Tg ischemic muscle (Supplemental immunoblots for Figure 6).

**Figure 7:**
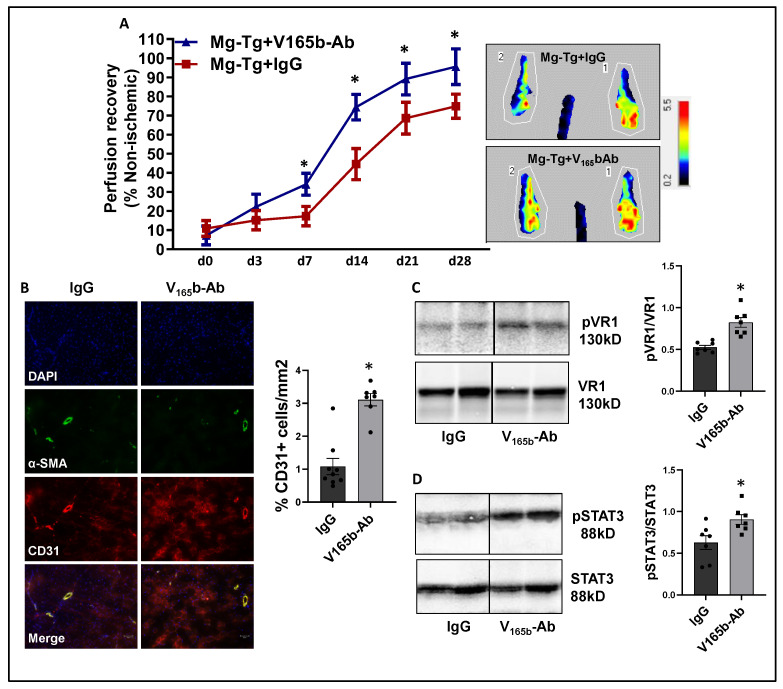
VEGF_165_b inhibition improves perfusion recovery in Mg-Tg mice by activating VEGFR1-STAT3 signaling in experimental PAD. (**A**) Laser Doppler microvascular perfusion imaging of Mg-Tg mice treated with IgG or V_165_b-Ab over the time course of recovery. *n* ≥ 7. Repeated Measures ANOVA with Dunnett’s post-test. * *p* < 0.05 considered significant at specific time points. (**B**) Immunohistochemical analysis of CD31 in Mg-Tg mice ischemic gastrocnemius muscle treated with IgG or V_165_b-Ab at day 28 post-HLI. *n* ≥ 7. Unpaired *t*-test. (**C**,**D**) Immunoblot analysis of pVR1/VR1, pSTAT3/STAT3 in Mg-Tg mice ischemic gastrocnemius muscle treated with IgG or V_165_b-Ab at day 3 post-HLI. *n* = 7. Unpaired *t*-test. * *p* < 0.05 considered significant. Data Mean ± SEM.

Laser Doppler showed that VEGF_165_b-Ab in Mg-Tg ischemic muscle significantly enhanced perfusion recovery (d7: IgG-17.3 ± 1.7 vs. V_165_b-Ab-34.0 ± 2.1; d14: IgG-44.6 ± 2.9 vs. V_165_b-Ab-74.4 ± 2.5; d21: IgG-68.6 ± 2.7 vs. V_165_b-Ab-89.1 ± 3.1; d28: IgG-74.8 ± 2.1 vs. V_165_b-Ab-95.6 ± 3.5) vs. IgG (Figure 7A). The immunohistochemistry of CD31 showed a significant increase in CD31+ cells (~3-fold) in Mg-Tg ischemic muscle treated with VEGF_165_b-Ab vs. IgG, indicating that VEGF_165_b inhibition can induce microvascular density despite a lack of NO bioavailability (Figure 7B). We next performed immunoblot analysis to determine whether enhanced perfusion and microvascular density are due to activation of VEGFR1-STAT3 signaling in VEGF_165_b-Ab-treated Mg-Tg ischemic muscle. Immunoblot analysis showed a significant increase in pVEGFR1_Y1333_ activation (~1.5-fold, Figure 7C) and pSTAT3 activation (~1.3-fold, Figure 7D) in VEGF_165_b-Ab-treated Mg-Tg ischemic muscle vs. IgG indicating that the activation of VEGFR1-STAT3 signaling by VEGF_165_b inhibition-induced microvascular density and promoted perfusion recovery in Mg-Tg ischemic muscle. Finally, we performed immunoblot analysis of VEGFR2 signaling intermediates, including pAKT and pERK in Mg-Tg ischemic muscle. VEGF_165_b inhibition in Mg-Tg ischemic muscle significantly decreased pAKT activation (~1.6-fold, Supplementary Figure S12A) but not pERK activation vs. IgG (Supplementary Figure S12B).

**VEGF_165_b inhibition induces an angiogenic response by activating VEGFR1 signaling in an in vitro diabetic-PAD model of impaired VEGFR2 signaling**. Previous studies have demonstrated that plasma levels of palmitic acid (PA) have a strong association with Type-2 diabetes patients [39,40] and inhibit AKT, ERK, and eNOS activation as well as a VEGF-A-induced tube-like formation on Matrigel [41,42,43]. Based on these data, we developed an in vitro model of diabetic-PAD, wherein HUVECs were challenged with HSS in the presence of palmitic acid (PA, as an in vitro model for diabetes) to determine an EC-specific role of VEGF_165_b-VEGFR1 signaling in regulating angiogenesis in a pathologically relevant in vitro model with impaired VEGFR2 signaling.

**Figure 8:**
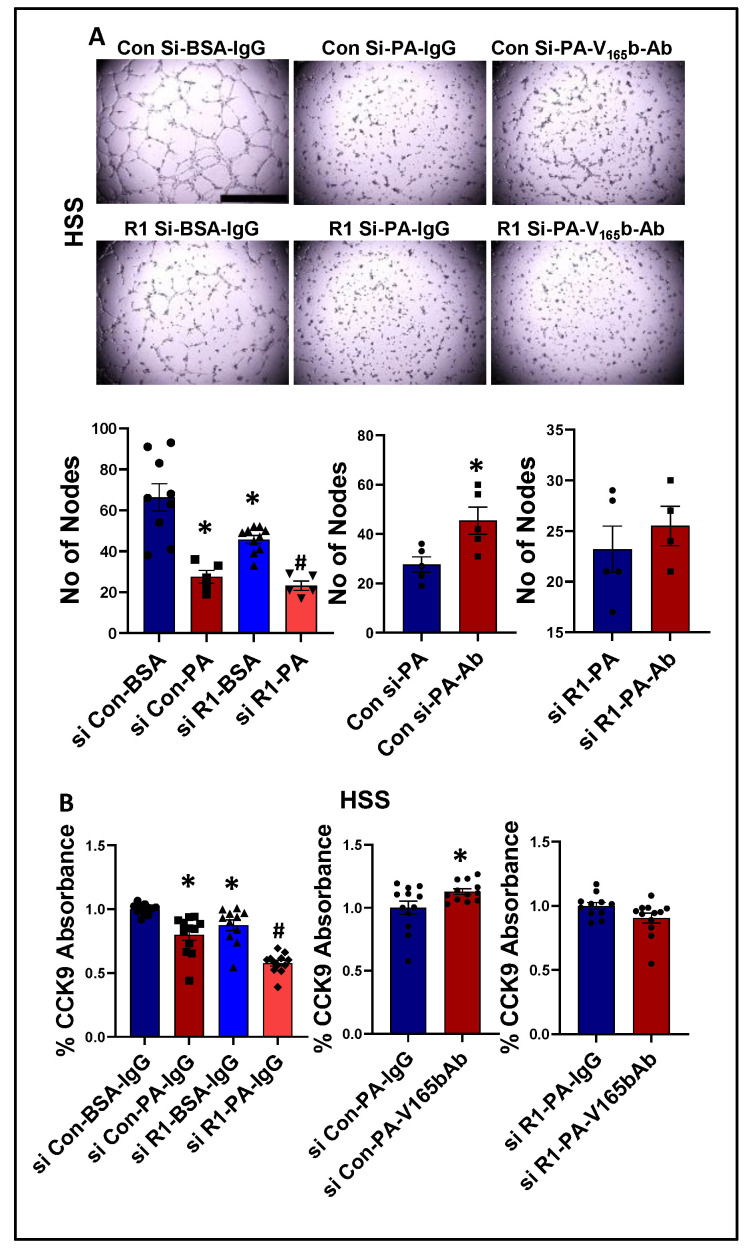
VEGF_165_b inhibition restores impaired angiogenic capacity and improves endothelial survival that is dependent on VEGFR1 in in vitro diabetic-PAD model: (**A**) In vitro tube formation assay of HUVECs transfected with non-targeting control siRNA (Con Si) or VEGFR1 siRNA (R1 Si), treated with BSA or BSA-Palmitic acid (500 μM) and IgG or V_165_b-Ab (10 μg/mL) for 24 h under HSS conditions, *n* ≥ 4. One-way ANOVA with Bonferroni select pair comparison. * indicates significantly different from Con Si-BSA-IgG, # indicates significantly different from R1 Si-PA-IgG. Unpaired *t*-test for IgG vs. V_165_b-Ab comparisons. Scale-100 um. (**B**) Cell proliferation/survival assay of HUVECs transfected with non-targeting control siRNA (Con Si) or VEGFR1 siRNA (R1 Si), treated with BSA or BSA-Palmitic acid and IgG or V_165_b-Ab for 24 h under HSS conditions, *n* ≥ 11. One-way ANOVA with Bonferroni select pair comparison * indicates significantly different from Con Si-BSA-IgG, # indicates significantly different from Con Si-PA-IgG and R1 Si-PA-IgG. Unpaired *t*-test for IgG vs. V_165_b-Ab comparisons. * *p* < 0.05 considered significant. Data Mean ± SEM.

We next determined whether PA impairs endothelial angiogenic capacity and VEGF_165_b inhibition can rescue the PA-induced angiogenic inhibition. Matrigel analysis showed a significant decrease in the angiogenic capacity in normal (~2.2-fold, Supplementary Figure S13A) and HSS (~2.4-fold, Figure 8A)-HUVECs treated with PA vs. BSA. VEGFR1-silencing (Supplementary Figure S14) further decreased the angiogenic capacity of PA-treated normal HUVECs (~3-fold, Supplementary Figure S13A). However, due to a severe angiogenic inhibition in PA-treated HSS-HUVECs, we were not able to observe a further inhibition in the angiogenic capacity of VEGFR1-silenced HSS-HUVECs treated with PA (Figure 8A). VEGF_165_b-Ab significantly increased the angiogenic capacity of PA treated normal (~1.5-fold, Supplementary Figure S13A) and HSS-HUVECs (~1.6-fold, Figure 8A) vs. IgG. VEGF_165_b-Ab did not affect the angiogenic capacity of VEGFR1-silenced normal or HSS-HUVECs treated with PA (Supplementary Figure S13A, Figure 8A), indicating that VEGFR1 is necessary for VEGF_165_b-Ab to rescue PA-induced angiogenic impairment. We further confirmed these findings by performing cell survival assays in in vitro diabetic-PAD models. PA treated normal (Supplementary Figure S13B) or HSS (Figure 8B) HUVECs showed a significant decrease in cell proliferation/survival vs. BSA. VEGFR1 silencing further decreased the angiogenic capacity of PA-treated normal and HSS-HUVECs (Supplementary Figure S13B, Figure 8B). While VEGF_165_b-Ab significantly increased the cell proliferation/survival in normal and HSS-HUVECs treated with PA; VEGF_165_b-Ab did not affect the cell proliferation/survival in VEGFR1 silenced normal or HSS-HUVECs treated with PA (Supplementary Figure S13B, Figure 8B).

**Figure 9:**
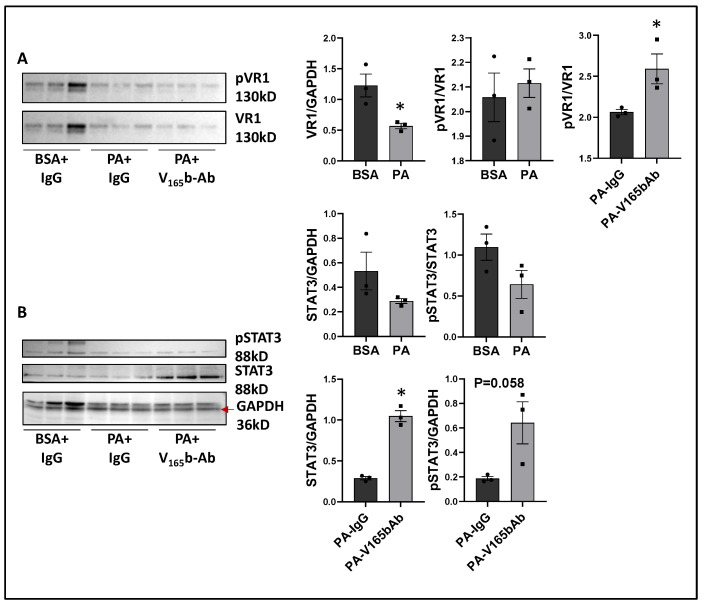
VEGF_165_b inhibition induces VEGFR1-STAT3 signaling in in vitro diabetic-PAD model: Immunoblot analysis of (**A**) pVR1/VR1, (**B**) pSTAT3/STAT3 in HSS-HUVECs treated with BSA + IgG, PA + IgG, PA + V_165_b-Ab. *n* = 3. Unpaired *t*-test. * *p* < 0.05 considered significant.

We next determined the signaling changes induced by PA in HSS-HUVECs and the signaling induced by VEGF_165_b-Ab in PA-treated HSS-HUVECs that resulted in increased angiogenesis. Immunoblot analysis showed no significant difference in VEGF_165_b vs. total VEGF-A levels between PA vs. BSA-treated HSS-HUVECs (Supplementary Figure S15). However, PA significantly decreased total VEGFR1 levels vs. BSA-treated HSS-HUVECs (~1.8-fold, Figure 9A). VEGF_165_b-Ab did not affect total VEGFR1 levels in PA-treated HSS-HUVECs but induced pVEGFR1_Y1333_ activation vs. IgG (*p* = 0.046, ~1.4-fold, Figure 9A). We next performed immunoblot analysis of pSTAT3 activation to confirm VEGFR1 signaling activation. No significant difference in STAT3 levels was observed between PA vs. BSA-treated HSS-HUVECs (Figure 9B). Interestingly, VEGF_165_b-Ab induced a dramatic upregulation of STAT3 levels and only a numeric but non-significant increase in pSTAT3 levels (*p* = 0.058) vs. IgG-treated PA-HSS-HUVECs (Figure 9B).

We next performed the immunoblot analysis of pVEGFR2_Y1175_, pAKT, and pERK activation to examine whether PA impairs VEGFR2 signaling in HSS-HUVECs; and whether VEGF_165_b-Ab modulates VEGFR2 signaling in PA-treated HSS-HUVECs. Immunoblot analysis of VEGFR2 showed a significant decrease in VEGFR2 levels in PA-treated HSS-HUVECs vs. BSA (Supplementary Figure S16A). VEGF_165_b-Ab did not affect total VEGFR2 levels or pVEGFR2_Y1175_ activation in PA-treated HSS-HUVECs vs. IgG. Immunoblot analysis of AKT showed no significant difference in AKT levels between PA- and BSA-treated HUVECs (Supplementary Figure S16B). VEGF_165_b-Ab did not affect pAKT activation in PA-treated HSS-HUVECs vs. IgG. The immunoblot analysis of ERK showed no significant difference in ERK levels in PA-treated HSS-HUVECs vs. BSA (Supplementary Figure S16C). However, a significant increase in pERK activation was observed between PA- vs. BSA-treated HSS-HUVECs. VEGF_165_b-Ab did not affect pERK activation in PA-treated HSS-HUVECs vs. IgG (Supplementary Figure S16C).

## 4. Discussion

Attempts to promote therapeutic angiogenesis in humans have been underway for almost two decades but the studies have been met with limited success. VEGF was one of the extensively studied agents [44]. Potential explanations for the lack of success with VEGF include inadequate dosing to activate VEGFR signaling in ischemic tissue, but the inability of humans to activate VEGFR2-Akt-eNOS-NO in an ischemic environment remains a plausible explanation. In a prior experimental PAD study, where the VEGFR2-Akt-eNOS-NO system could be activated, we demonstrated that VEGF_165_b was upregulated and acted as an anti-angiogenic agent via VEGFR1, but not via VEGFR2 [8,15]. Here we demonstrate that a monoclonal antibody targeting VEGF_165_b [8,9] was able to promote therapeutic angiogenesis in systems where NO production is impaired and/or its bioavailability is reduced. These data fill important gaps in the consideration of therapeutics for PAD.

In mice models, and in humans, diabetes further impairs arteriogenesis and angiogenesis in PAD muscle compared with PAD muscle alone [18,23]. Attenuated VEGFR2 signaling in murine Type-2 diabetic (high-fat diet) ischemic muscle (T2D-HLI) has been shown to play a causal role in inhibiting perfusion recovery [17,18,45]. We hypothesized that an induction in VEGF_165_b isoform in T2D-HLI muscle plays a causal role in inhibiting angiogenesis in T2D-HLI. We have previously shown that ischemia induces anti-angiogenic VEGF_165_b levels in endothelial cells challenged with hypoxia serum starvation vs. normal growth conditions; and in endothelial cells from ischemic muscle vs. non-ischemic muscle [8]. However, treating endothelial cells with Palmitate and hypoxia serum starvation as a model for T2D-PAD did not further increase VEGF_165_b levels. Accordingly, no significant differences in either total VEGF-A or VEGF_165_b levels were observed between normal vs. T2D ischemic muscle. However, the fraction of VEGF_165_b bound to VEGFR1 was significantly higher resulting in the loss of VEGFR1 activation in the ischemic muscle from T2D-PAD mice vs. ischemic muscle from mice on normal chow. Removing the inhibitory effect of VEGF_165_b bound to VEGFR1 allowed VEGFR1-STAT3 signaling activation that resulted in increased angiogenesis and perfusion recovery in T2D-HLI muscle. A previous study by Kikuchi et al. showed that VEGF_165_b inhibition increased both collateral (assessed as SMA+ vessels) and microvascular density in diabetic mouse models, including the high fat-high sucrose diet model and Ob-Ob mice model [9]. Taken together, VEGF_165_b inhibition induces both arteriogenesis and angiogenesis in ischemic diabetic muscle to enhance perfusion recovery. The ability of VEGFR1-STAT3 signaling to induce angiogenesis and perfusion recovery in the T2D-HLI model presented a critical role of VEGFR1-STAT3 signaling to induce therapeutic angiogenesis in PAD.

**Figure 10:**
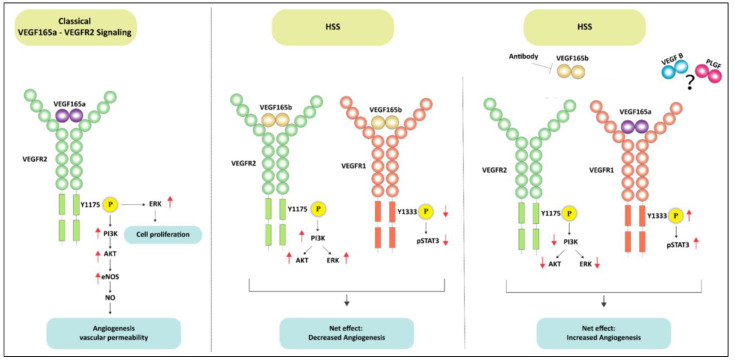
Schematic of VEGFR1 vs. VEGFR2 signaling activation by VEGF_165_b in ischemic endothelium. The left panel shows the classical VEGF_165_a induced VEGFR2 signaling pathway in ECs. The middle panel demonstrates that VEGF_165_b treatment induces VEGFR2-AKT-ERK signaling but inhibits VEGFR1-STAT3 signaling in ischemic ECs. Despite activating VEGFR2 signaling, the inhibition of VEGFR1 signaling resulted in angiogenic inhibition in ischemic ECs treated with VEGF_165_b. In the right panel, antibody-mediated VEGF_165_b inhibition increased the bioavailability of VEGF_165_a to VEGFR1 (due to its 10X higher binding affinity to VEGFR1 over VEGFR2) and activated VEGFR1-STAT3 signaling but resulted in the loss of VEGFR2 signaling activation. Activating VEGFR1-STAT3 signaling-induced ischemic angiogenesis independent of VEGFR2 signaling.

Previous reports have shown that VEGF_165_b, an alternatively spliced VEGF-A isoform blocks VEGF-A-induced angiogenesis by inhibiting VEGFR2 activation. On the contrary VEGF_165_b has been shown to be a weak activator of VEGFR2 albeit under normal growth conditions [12,13,33,35]. Consistently, our results show that VEGF_165_b induces VEGFR2 and downstream AKT-ERK-eNOS signaling to a similar extent as VEGF_165_a both under normal growth conditions, and when challenged with HSS, an in vitro model used to simulate the PAD muscle that is deprived of oxygen and growth factors [3,8,15]. Despite its ability to activate VEGFR2, the angiogenic capacity of VEGF_165_b is lower vs. VEGF_165_a indicating that VEGF_165_b inhibits angiogenesis independent of VEGFR2 in ischemic vasculature. Accordingly, VEGF_165_b inhibition decreased VEGFR2 signaling in HSS-HUVECs, indicating that VEGF_165_b is a potent activator of VEGFR2. A schematic of these data is presented in Figure 10. 

To confirm the role of VEGF_165_b inhibition in activating VEGFR1 in T2D-PAD vasculature, we developed an in vitro model of T2D-PAD. Diabetes is one of the most important risk factors for amputation in PAD patients [46]. Microvascular abnormalities and impaired angiogenesis result in ulcers and decreased wound healing in patients with PAD and diabetes [47]. Hence, we wanted to establish an in vitro model for diabetic-PAD. Previous studies have shown that palmitic acid induces reactive oxygen species, induces cell death, inhibits cell proliferation, and impairs angiogenesis in vitro [48,49]. Multivariate regression analysis of the data collected from the Insulin Resistance Atherosclerosis Study (IRAS) showed a strong association of palmitic acid with Type-2 diabetes risk independent of insulin sensitivity [50]. Furthermore, Reynoso et al. [51] have suggested that high palmitic acid levels lead to insulin resistance [51]. Taking these studies into consideration, we have employed a treatment where ECs are treated with starvation medium supplemented with palmitic acid followed by subjecting them to hypoxia. We hypothesized that this combinatorial treatment will be closer in vitro model for diabetic-PAD. Consistent with our hypothesis, ECs treated with palmitic acid showed a significant loss in their angiogenic capacity vs. normal controls; and ECs treated with serum starvation + palmitic acid under hypoxic conditions showed further loss of angiogenic capacity vs. serum starvation + hypoxia. Inhibiting VEGF_165_b in the context of this in vitro T2D-PAD model was able to rescue impaired angiogenesis. More importantly, VEGF_165_b inhibition induced VEGFR1 activation without affecting VEGFR2 activation or downstream signaling. 

To confirm whether VEGFR1 activation can induce therapeutic angiogenesis and perfusion independent of NO, we used eNOS-KO mice [19] and Myoglobin-transgenic mice [20,21] in the HLI model. Interestingly, while VEGF-A levels are significantly lower in eNOS-KO mice ischemic muscle; VEGF-A levels are significantly increased in Myoglobin-transgenic mice vs. their respective controls. NO has been shown to regulate VEGF-A synthesis [20,52]. In eNOS-KO ischemic muscle, a severe decrease in NO production could have resulted in decreased VEGF-A levels. Whereas in Myoglobin-transgenic mice, scavenging of NO by skeletal muscle, could have resulted in higher VEGF-A production/levels by skeletal muscle cells. Interestingly, while VEGF_165_b inhibition-induced ERK activation without changes in AKT activation in eNOS-KO ischemic muscle, VEGF_165_b inhibition in Mg-Tg ischemic muscle-inhibited AKT activation with no changes in ERK suggesting distinct VEGFR1 signaling events are activated post-VEGF_165_b inhibition across these mouse models.

## 5. Conclusions

Amputations due to PAD in addition to other cardiovascular risk factors increase the acute mortality rate to ~30% and decrease the 5-year prognostic survival rate to less than 30%, clearly indicating that there is a great need for identifying new therapeutic strategies to treat PAD [1,3,46]. Our current study shows that ischemic muscle revascularization can be achieved with VEGFR1 activation rather than VEGFR2-NO signaling. However, the ability of VEGFR1 to revascularize the ischemic muscle is dependent on inhibiting VEGF_165_b, a selective and potent inhibitor of VEGFR1. The ability of VEGFR1 activation to enhance perfusion even when eNOS-NO signaling is impaired demonstrates the translational potential of targeting VEGF_165_b to induce VEGFR1-mediated perfusion relief in PAD, where targeting VEGFR2/NO signaling has failed.

## 6. Highlights

- Anti-angiogenic VEGF_165_b activates VEGFR2-AKT-ERK-eNOS-NO signaling in ischemic vasculature with no pro-angiogenic effects.
- Antibody-mediated depletion of VEGF_165_b allowed VEGFR1-STAT3 activation to induce angiogenesis and promote perfusion recovery in preclinical PAD models, where VEGFR2-eNOS-NO signaling is impaired.
- Inhibiting VEGF_165_b in a Type-2 diabetic-PAD model induced VEGFR1-STAT3 signaling activation to promote perfusion recovery
- Blocking VEGF_165_b in eNOS-KO mice induced VEGFR1-STAT3 signaling to decrease necrosis incidence and severity in preclinical PAD
- Targeting VEGF_165_b promoted therapeutic angiogenesis where eNOS-NO signaling is impaired, demonstrating the translational potential of inducing VEGFR1-mediated perfusion relief in PAD, where targeting VEGFR2/NO signaling has failed.

## Data Availability

The data presented in this study are available on request from the corresponding author.

## Supplementary Materials

The following supporting information can be downloaded at: https://www.mdpi.com/article/10.3390/cells11172676/s1, Figure S1: Total VEGF-A levels in normal vs. HSS HUVECs. Figure S2: VEGF_165_a and VEGF_165_b differentially regulate total VEGFR2, AKT, and ERK levels. Figure S3: HSS decreases VEGF-B and PLGF levels in HUVECs. Figure S4: VEGF-A and VEGF_165_b levels in normal chow and T2D-PAD model. Figure S5: T2D-PAD mice have lower VEGFR2 activation in ischemic muscle. Figure S6: VEGF_165_b inhibition doesn’t induce VEGFR2-eNOS signaling in the T2D-PAD model. Figure S7: STAT3 inhibition decreases AKT activation in normal and HSS HUVECs. Figure S8: VEGF_165_b-inhibition inhibits apoptosis T2D-PAD model. Figure S9: VEGF_165_b, VEGF-A levels, and VEGFR1 activation in eNOS-KO mice in experimental PAD. Figure S10: VEGF_165_b inhibition induced ERK but not AKT activation in eNOS-KO mice ischemic muscle. Figure S11: VEGF_165_b, VEGF-A levels, and VEGFR1 activation in Myoglobin-transgenic (Mg-Tg) mice in experimental PAD. Figure S12: VEGF_165_b inhibition decreased AKT activation in Mg-Tg mice ischemic muscle. Figure S13: VEGFR1 silencing blocked the ability of VEGF_165_b-Ab to enhance the angiogenic capacity of endothelial cells in the in vitro T2D-PAD model. Figure S14: qPCR confirming VEGFR1 silencing in HUVECs transfected with VEGFR1 siRNA. Figure S15: Palmitic acid did not induce significant differences in VEGF_165_b vs. VEGF-A levels in HSS HUVECs. Figure S16: VEGF_165_b-inhibition does not induce VEGFR2-signaling in in vitro diabetic-PAD model.

## Abbreviations

Ab	Antibody
AKT	Protein Kinase-B
EC	Endothelial cells
eNOS	endothelial Nitric Oxide Synthase
ERK	Extracellular signal-regulated kinase
HFD	High Fat Diet
HSS	Hypoxia serum starvation
HUVEC	Human Umbilical Vein Endothelial Cells
IgG	Immunoglobulin G
Mg	Tg-Myoglobin-transgenic
MVEC	Mouse skeletal muscle endothelial cells
NO	Nitric oxide
PAD	Peripheral Artery Disease
PA	palmitic acid
PLGF	Placental Growth Factor
STAT3	Signal Transducer and Activator of Transcription 3
T2D	Type-2-diabetes
VEGF	Vascular Endothelial Growth factor
VEGFR	Vascular Endothelial Growth Factor Receptor
WT	Wild type
