# Supplementary material for: Targeting Anti-Angiogenic VEGF165b–VEGFR1 Signaling Promotes Nitric Oxide Independent Therapeutic Angiogenesis in Preclinical Peripheral Artery Disease Models
Source: Cells. 2022 Aug 28;11(17):2676. doi: 10.3390/cells11172676 (PMC9454804; doi:10.3390/cells11172676)

Figure S1

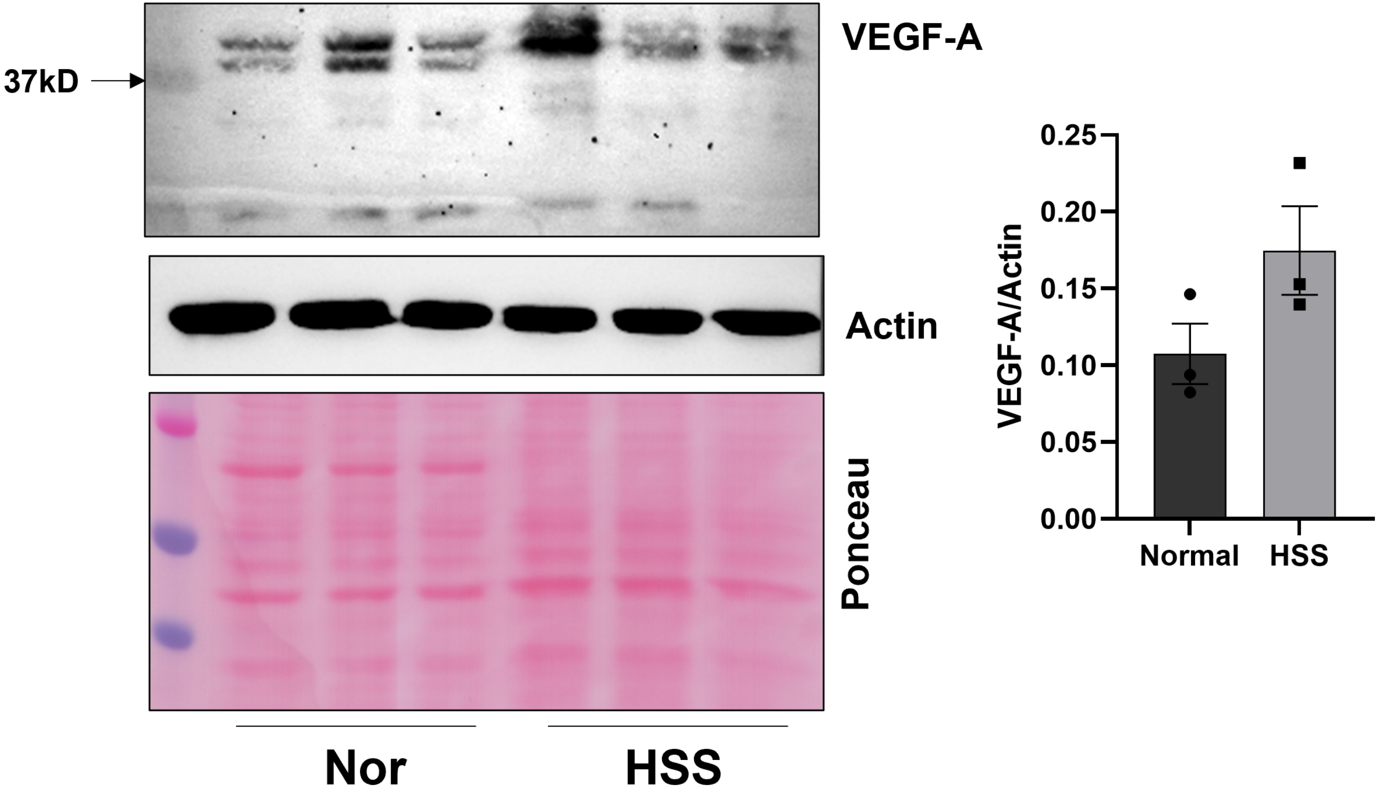

<u>Figure S1</u>. Total VEGF-A levels in normal vs. HSS HUVECs. Western blot analysis of VEGF-A using a pan-VEGF-A antibody that recognizes all the VEGF-A isoforms (Pro- and Anti-Angiogenic VEGF-A isoforms) in normal (Nor) and HSS (Hypoxia Serum Starved, for 24h) HUVECs. n=3. Unpaired T-Test. *P<0.05 considered significant. Data Mean±SEM

Figure S2: HSS HUVECs

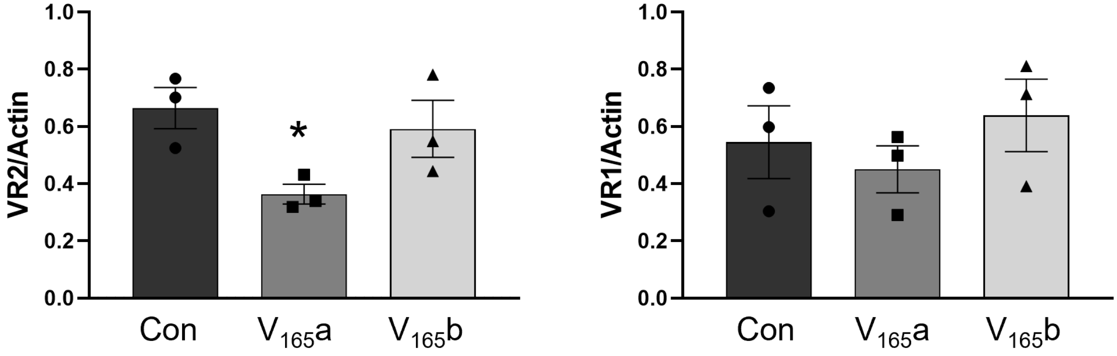

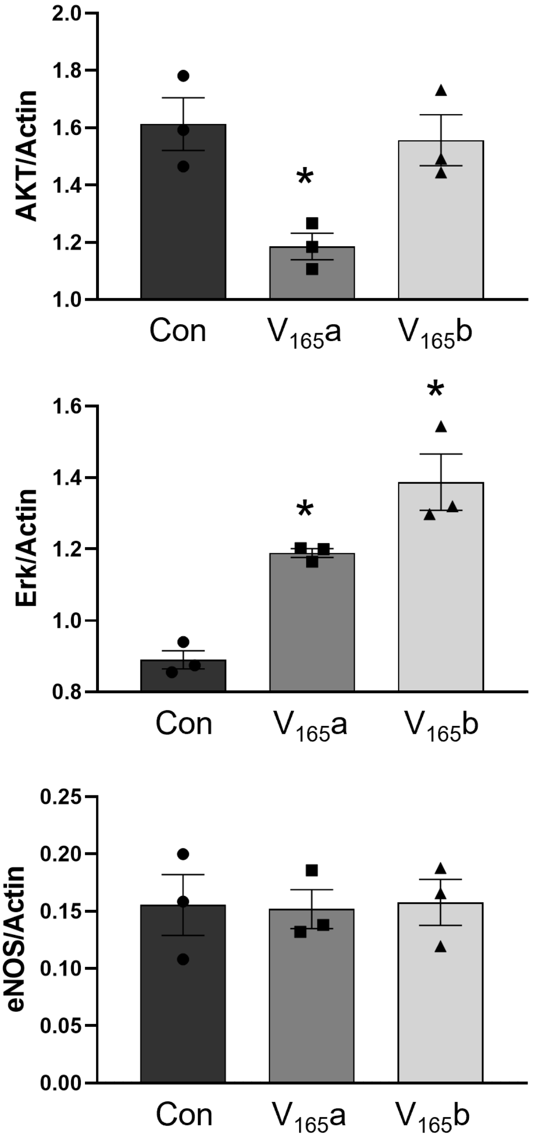

<u>Figure S2</u>. $VEGF_{165}a$ and $VEGF_{165}b$ differentially regulate total VEGFR2, AKT and ERK levels: Western blot analysis of VEGFR2 (VR2), AKT, ERK, eNOS, VR1 in HSS HUVECs treated with $VEGF_{165}a$ ($V_{165}a$) or $VEGF_{165}b$ ($V_{165}b$). n=3, One-way ANOVA with Bonferroni select pair comparison. *$P<0.05$ considered significant. Data Mean±SEM

Figure S3

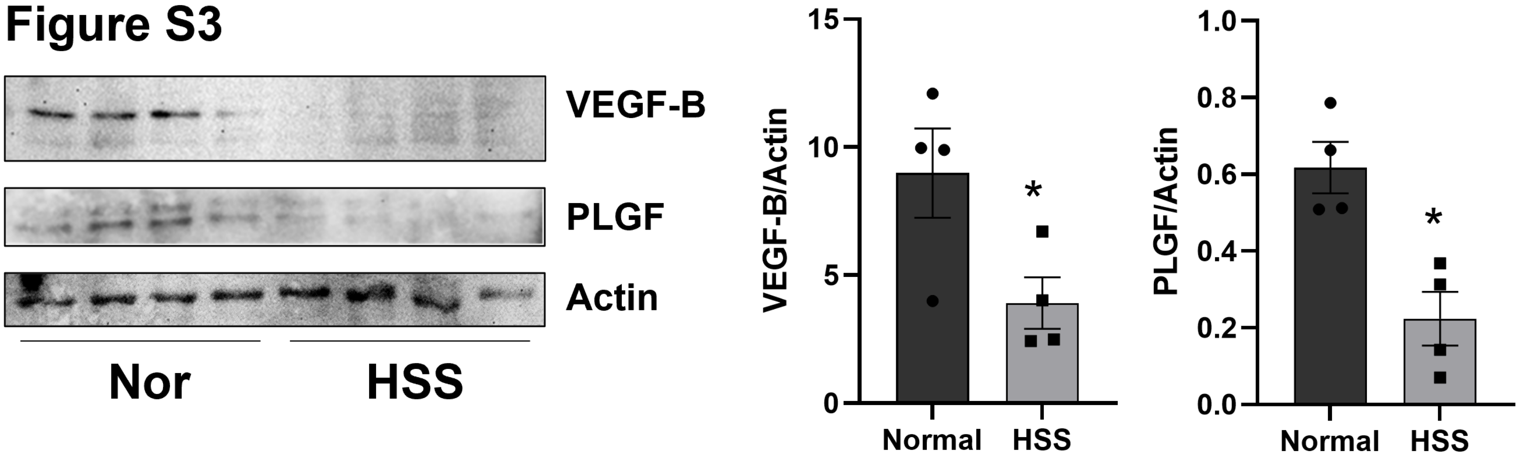

Figure S3. HSS decreases VEGF-B and PLGF levels in HUVECs: Western blot analysis of VEGF-B and PLGF in normal and HSS HUVECs. n=4, Unpaired T-test. *P<0.05 considered significant. Data Mean±SEM

Figure S4

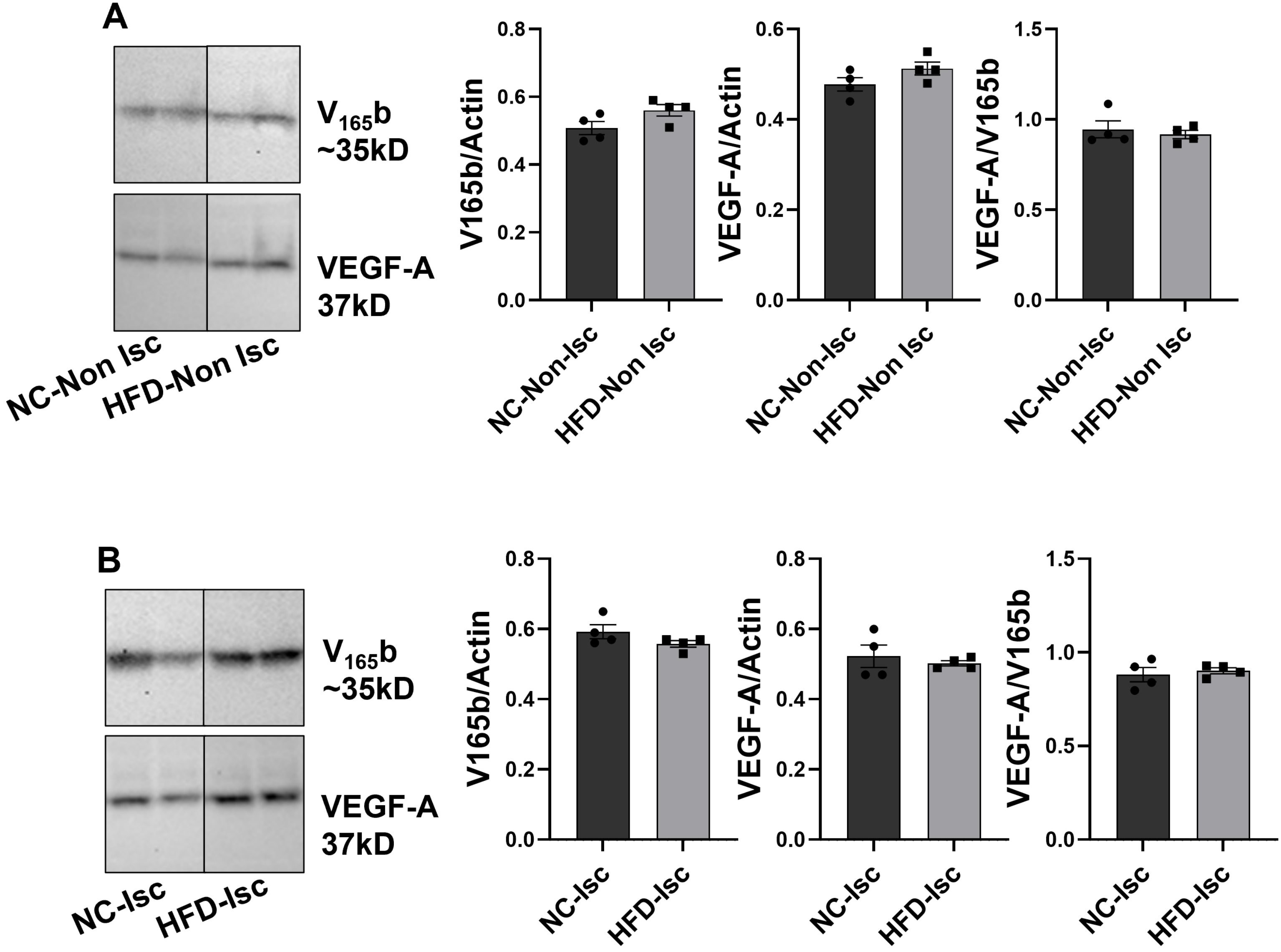

Figure S4. VEGF-A and $VEGF_{165}b$ levels in normal chow and T2D-PAD model. Immunoblot analysis of $VEGF_{165}b$ ($V_{165}b$) and total VEGF-A expression in A) non-ischemic gastrocnemius muscle (Non-Isc) of C57BL/6 mice on normal chow (NC) or high-fat diet (HFD). n=4, Unpaired T-test, and B) ischemic gastrocnemius muscle (Isc) of C57BL/6 mice on normal chow (NC) or high-fat diet (HFD) at day-3 post-HLI. n=4, Unpaired T-test. *P<0.05 considered significant. Data Mean±SEM.

Figure S5: T2D-HLI

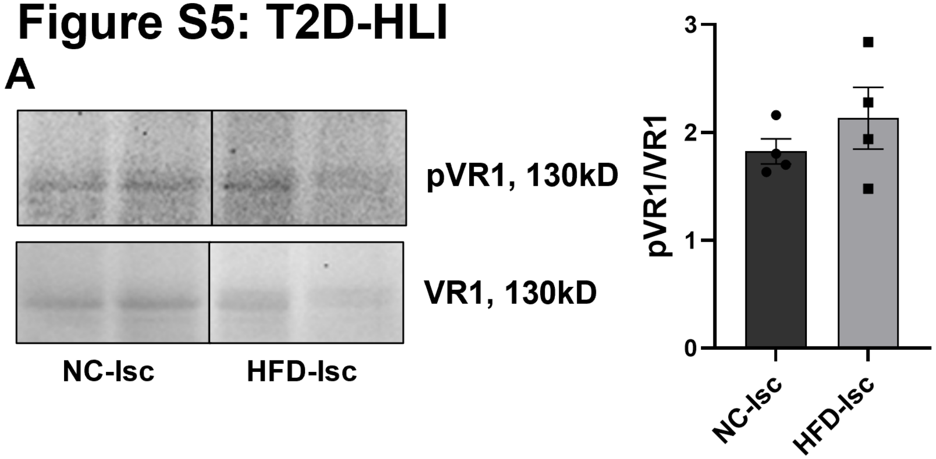

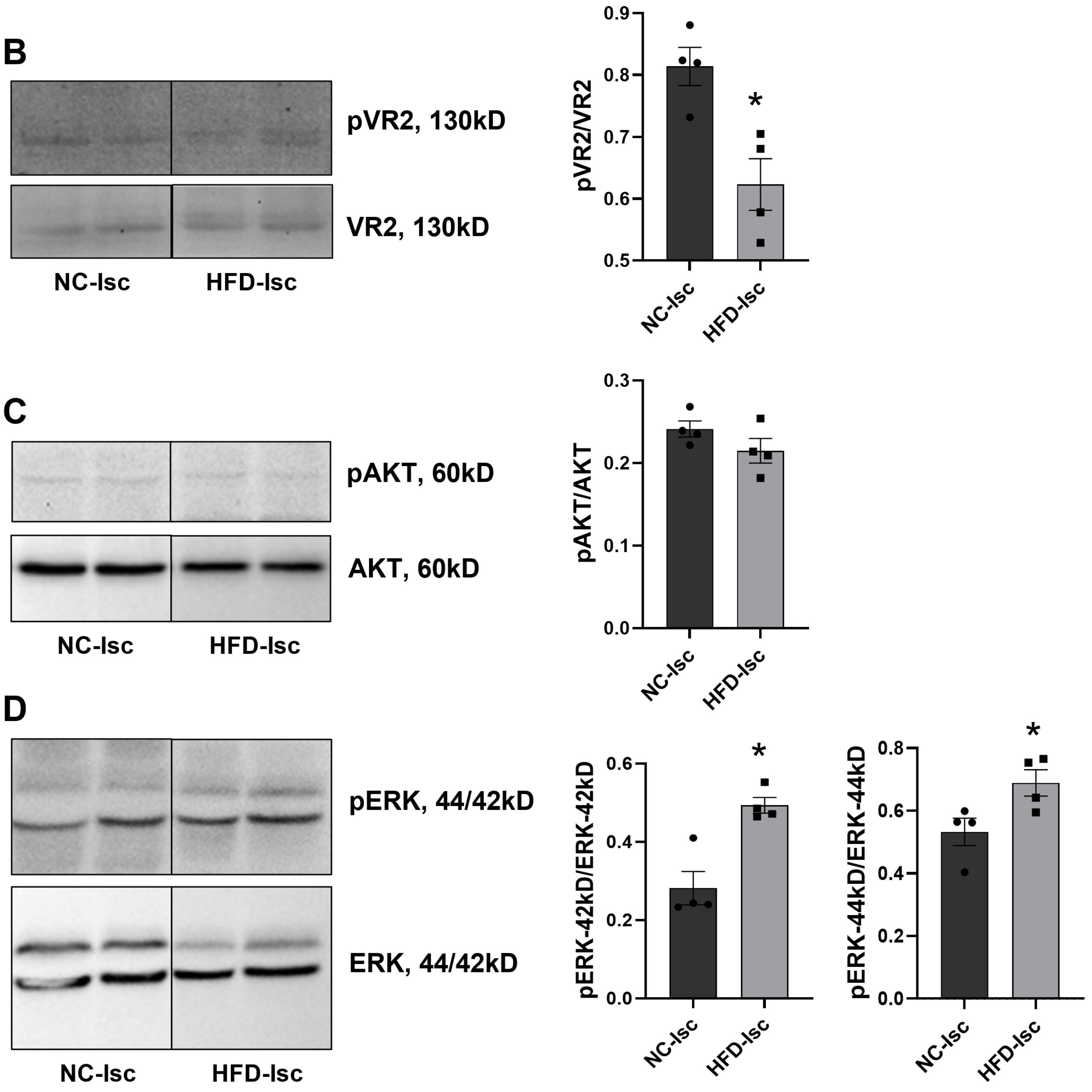

<u>Figure S5</u>. T2D-PAD mice have lower VEGFR2 activation in ischemic muscle: A) pVEGFR1$_{Y1333}$ (pVR1), VEGFR1 (VR1); B) pVEGFR2, VEGFR2; C) pAKT, AKT, D) pERK, ERK in normal chow (NC) and high-fat diet (HFD) fed ischemic muscle (Isc). n=4, Unpaired T-test. *P<0.05 considered significant. Data Mean±SEM

Figure S6: T2D-HLI

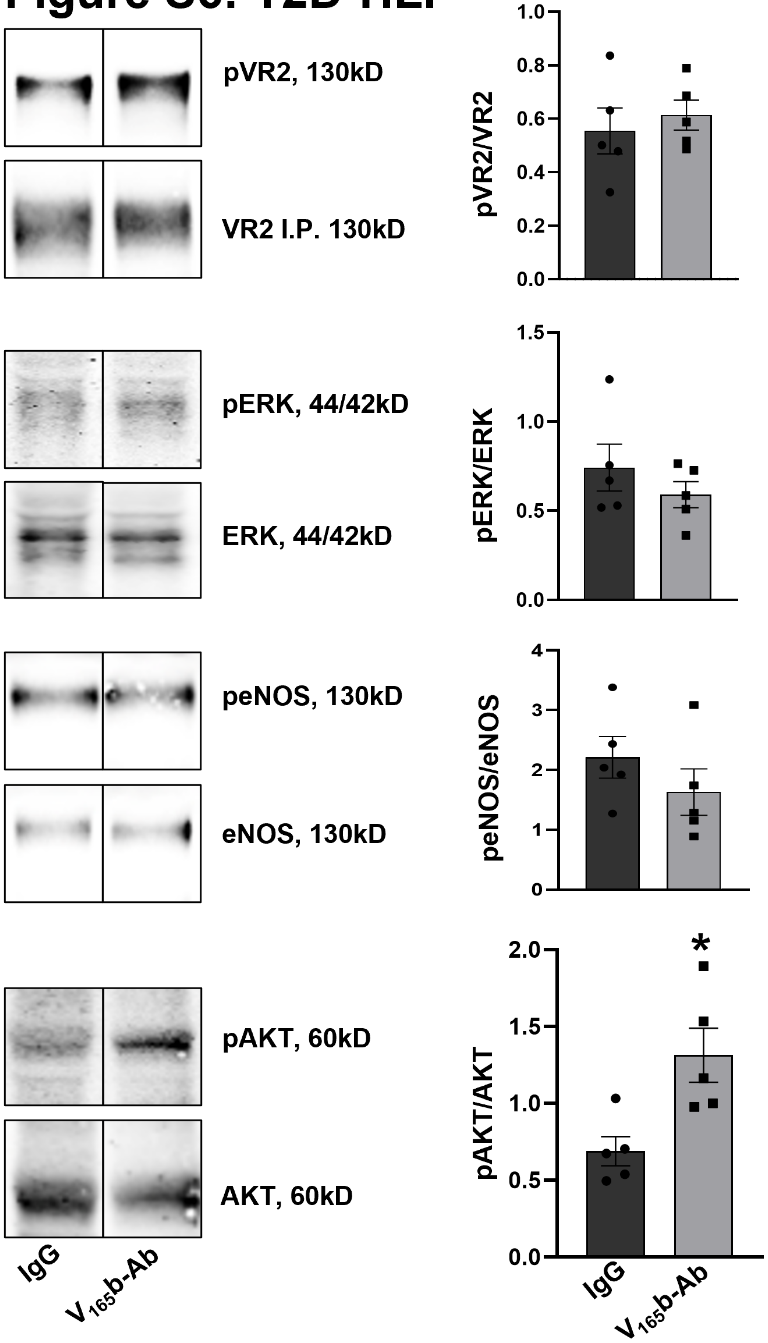

Figure S6. VEGF$_{165}$b inhibition doesn't not induce VEGFR2-eNOS signaling in T2D-PAD model. Western blot analysis of pVEGFR2, VEGFR2; pERK, ERK; peNOS, eNOS; pAKT, AKT in IgG or VEGF$_{165}$b-Ab treated high-fat diet fed ischemic muscle (Type-2 diabetic hind limb ischemia modes (T2D-HLI). n=5, Unpaired T-test. *P<0.05 considered significant. Data Mean±SEM

Figure S7

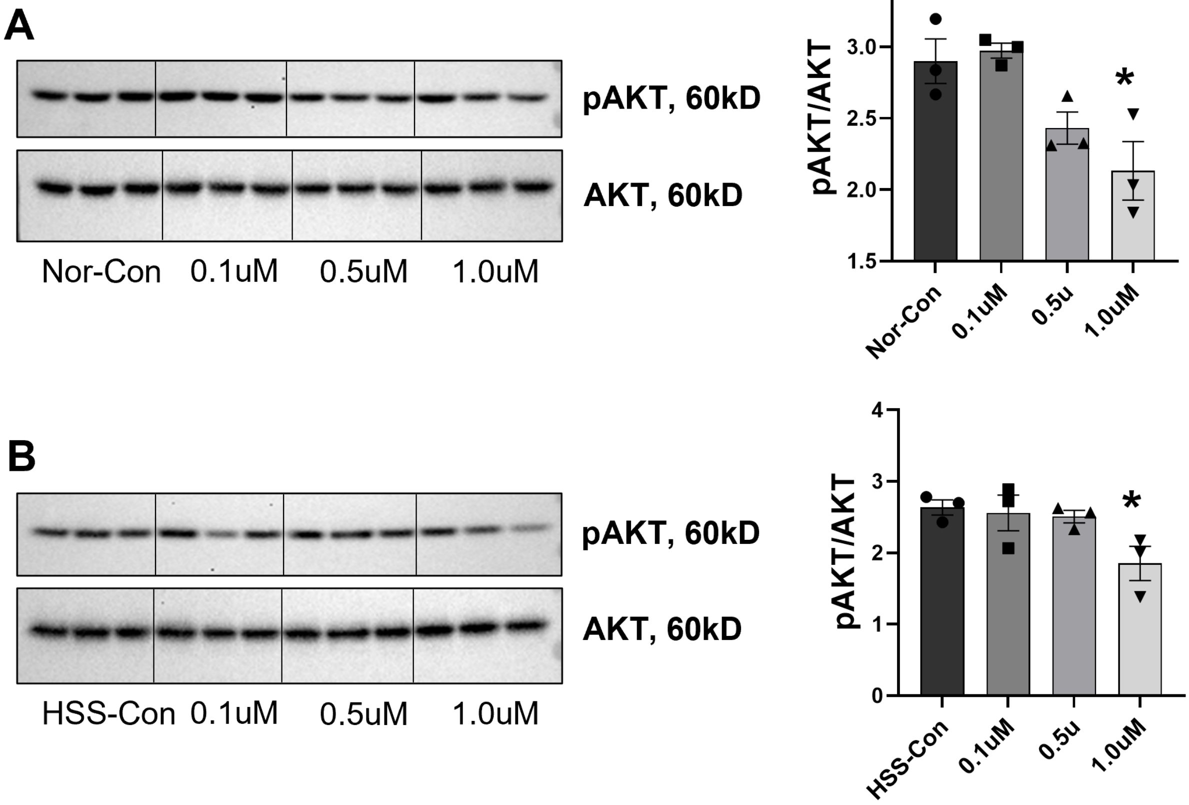

<u>**Figure S7**</u>. STAT3 inhibition decreases AKT activation in normal and HSS HUVECs: Western blot analysis of pAKT, AKT in A) normal or B) HSS HUVECs treated with 0.1μM, 0.5 μM. 1.0 μM STATTIC for 24h. n=3, One Way ANOVA with Dunnett's post-test. *P<0.05 considered significant. Data Mean±SEM

Figure S8

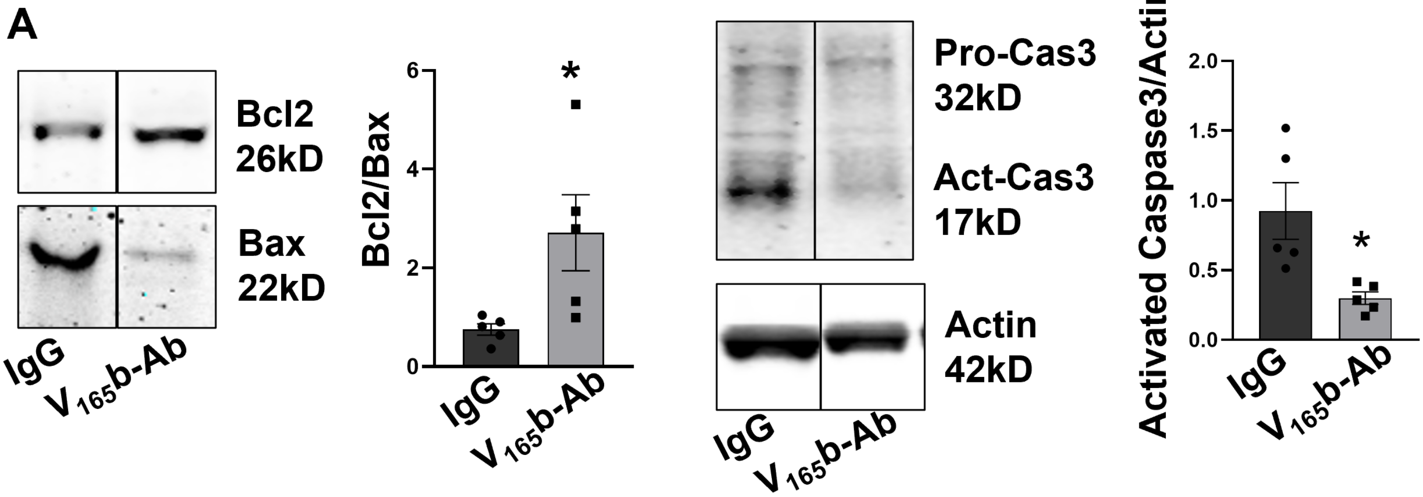

Figure S8. VEGF$_{165}$b-inhibition inhibits apoptosis T2D-PAD model: Immunoblot analysis of apoptotic protein expression levels including Bcl2, Bax, and activated Caspase-3 in HFD-ischemic gastrocnemius muscle treated with IgG or V$_{165}$b-Ab at day-3 post-HLI. n=5. Unpaired T-test. *P<0.05 considered significant. Data Mean±SEM

Figure S9

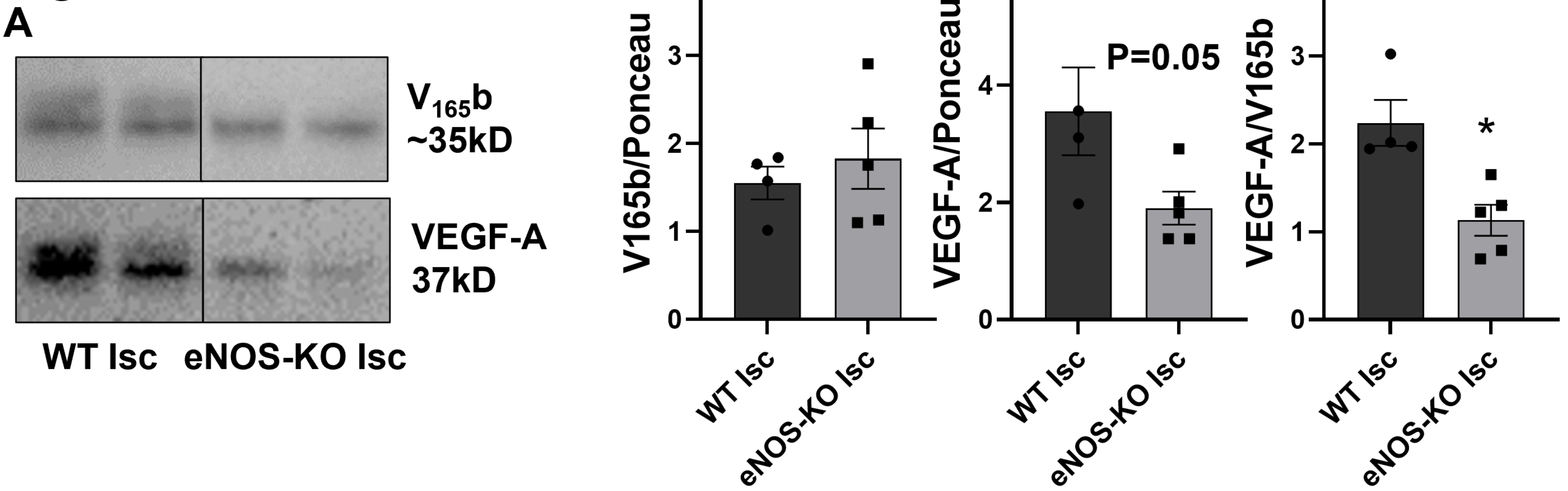

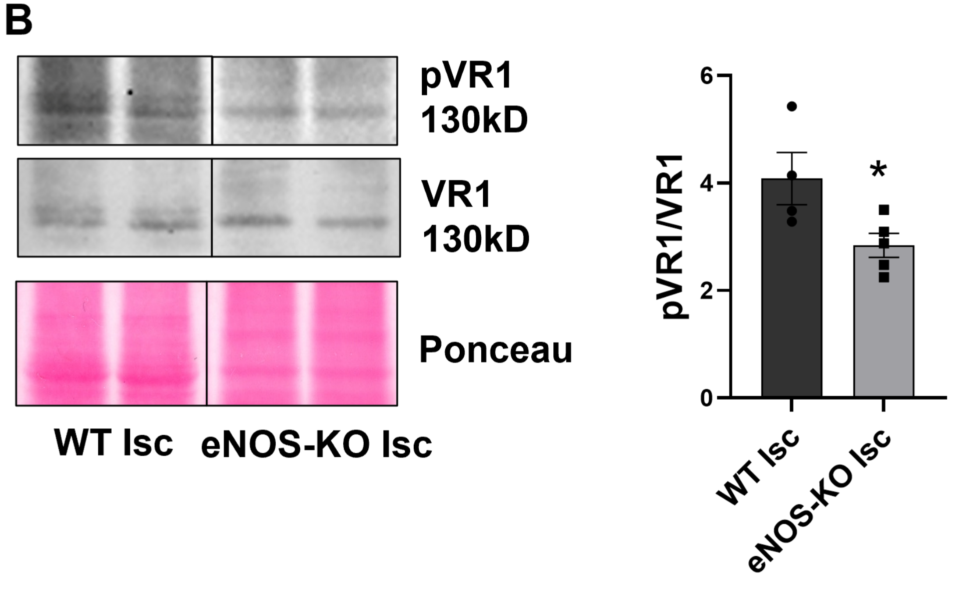

<u>Figure S9</u>. VEGF$_{165}$b, VEGF-A levels, and VEGFR1 activation in eNOS-KO mice in experimental PAD. A) Immunoblot analysis of VEGF$_{165}$b (V$_{165}$b) and total VEGF-A levels; pVR1 and VR1 in wild type controls and eNOS-KO mice ischemic gastrocnemius muscle at day-3 post-HLI. n≥5, Unpaired T-test. *P<0.05 considered significant. Data Mean±SEM

Figure S10: eNOS-KO-HLI

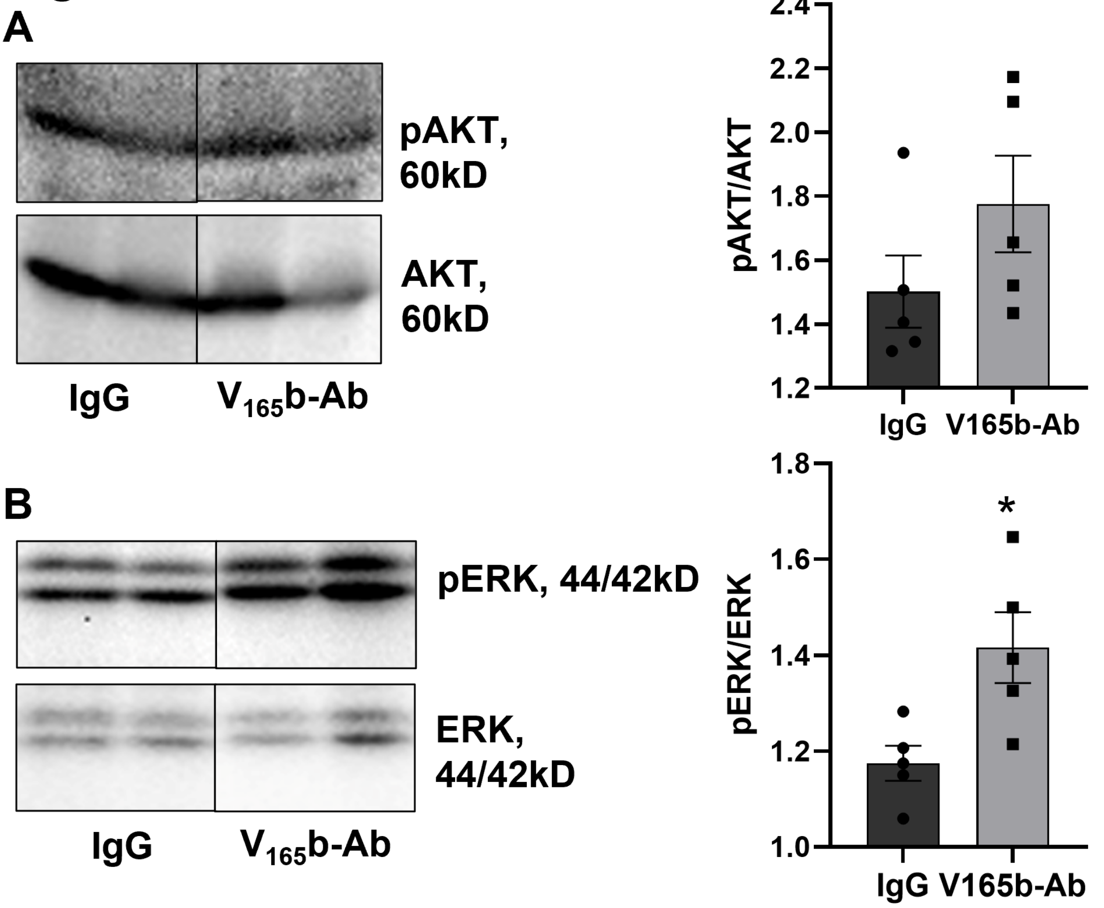

<u>Figure S10.</u> $VEGF_{165}b$ inhibition induced ERK but not AKT activation in eNOS-KO mice ischemic muscle: Western blot analysis of A) pAKT, AKT and B) pERK, ERK in IgG or $VEGF_{165}b$-Ab treated eNOS-KO mice ischemic muscle. n=5, Unpaired T-test. *P<0.05 considered significant. Data Mean±SEM

Figure S11

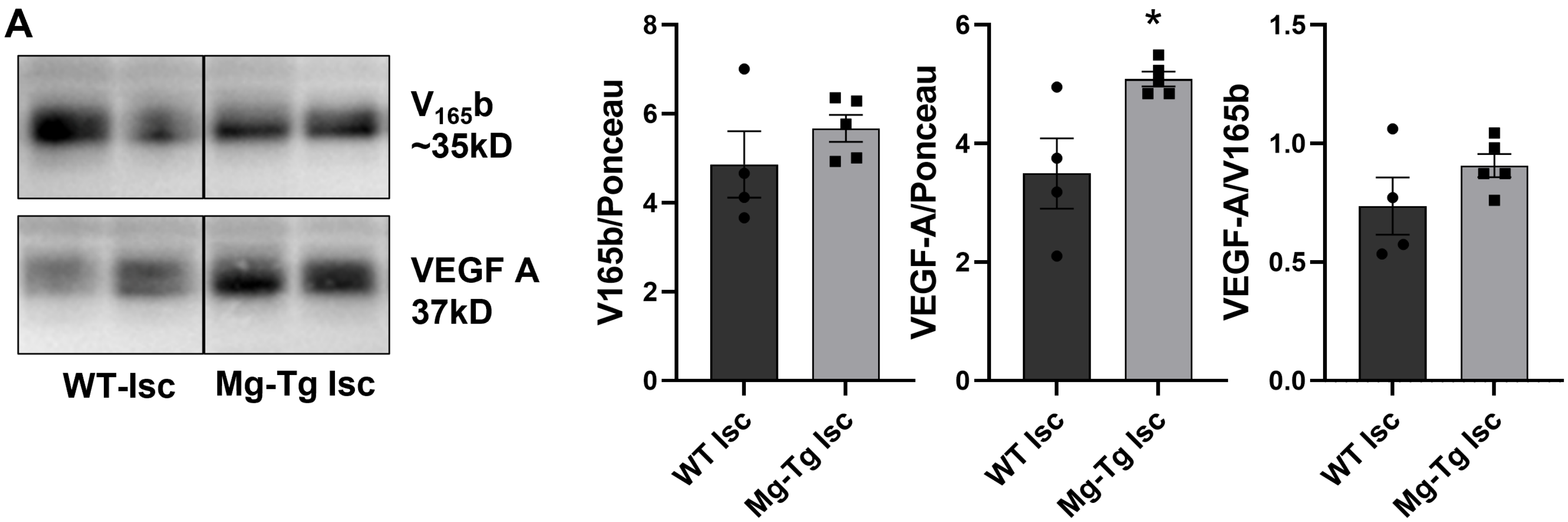

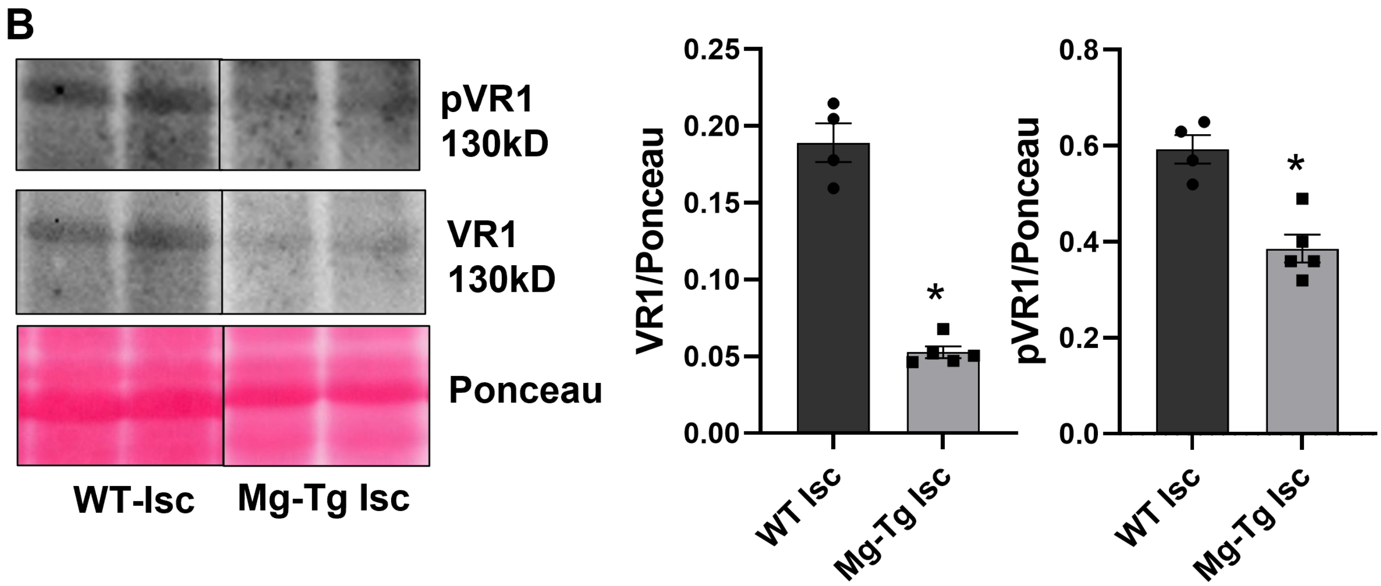

Figure S11. $VEGF_{165}b$, VEGF-A levels, and VEGFR1 activation in Myoglobin-transgenic (Mg-Tg) mice in experimental PAD. Immunoblot analysis of A) $VEGF_{165}b$ ($V_{165}b$) and total VEGF-A levels; B) pVR1 and VR1 in wild type controls and Mg-Tg mice ischemic gastrocnemius muscle at day-3 post-HLI. n≥4, Unpaired T-test. *P<0.05 considered significant. Data Mean±SEM

Figure S12: Mg-Tg-HLI

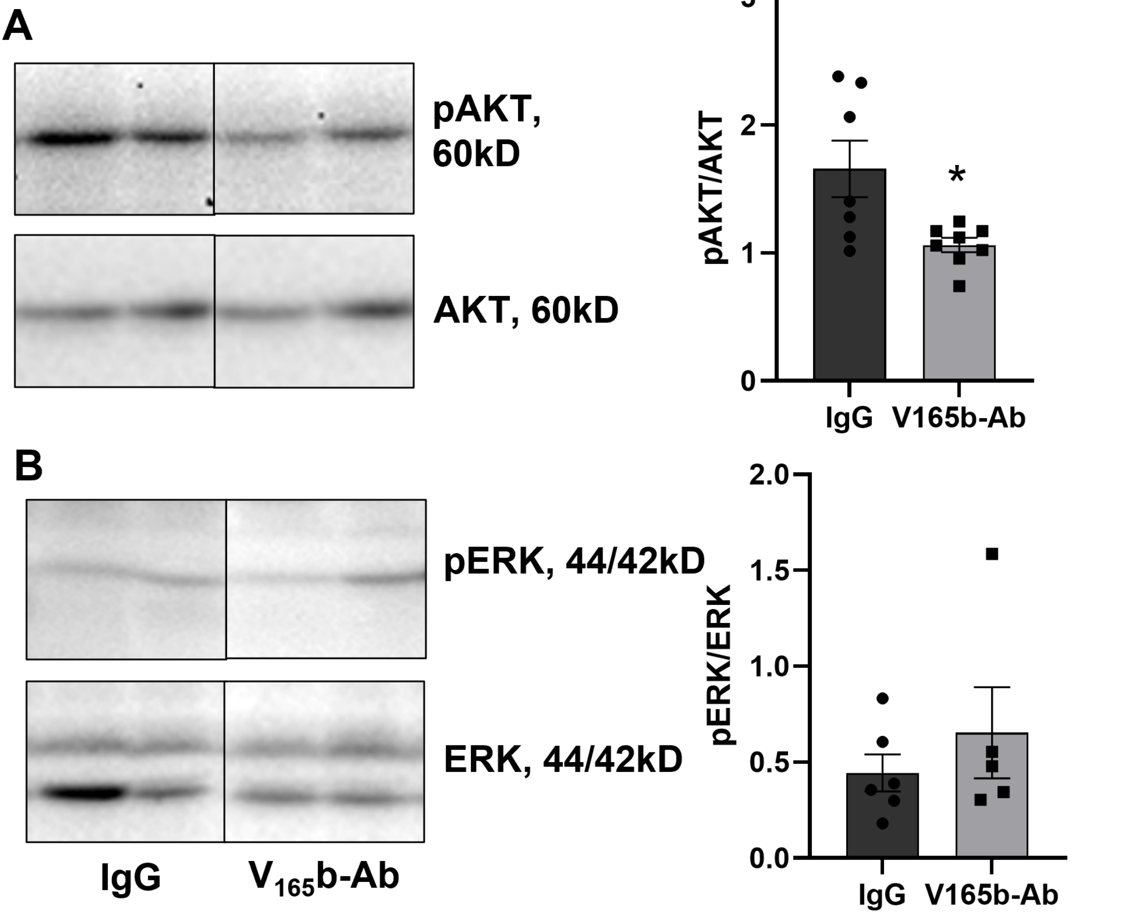

<u>Figure S12</u>. $VEGF_{165}b$ inhibition decreased AKT activation in Mg-Tg mice ischemic muscle: Western blot analysis of A) pAKT, AKT and B) pERK, ERK in IgG or $VEGF_{165}b$-Ab treated Mg-Tg mice ischemic muscle. n=7, Unpaired T-test. $^{*}P<0.05$ considered significant. Data Mean±SEM

Figure S13

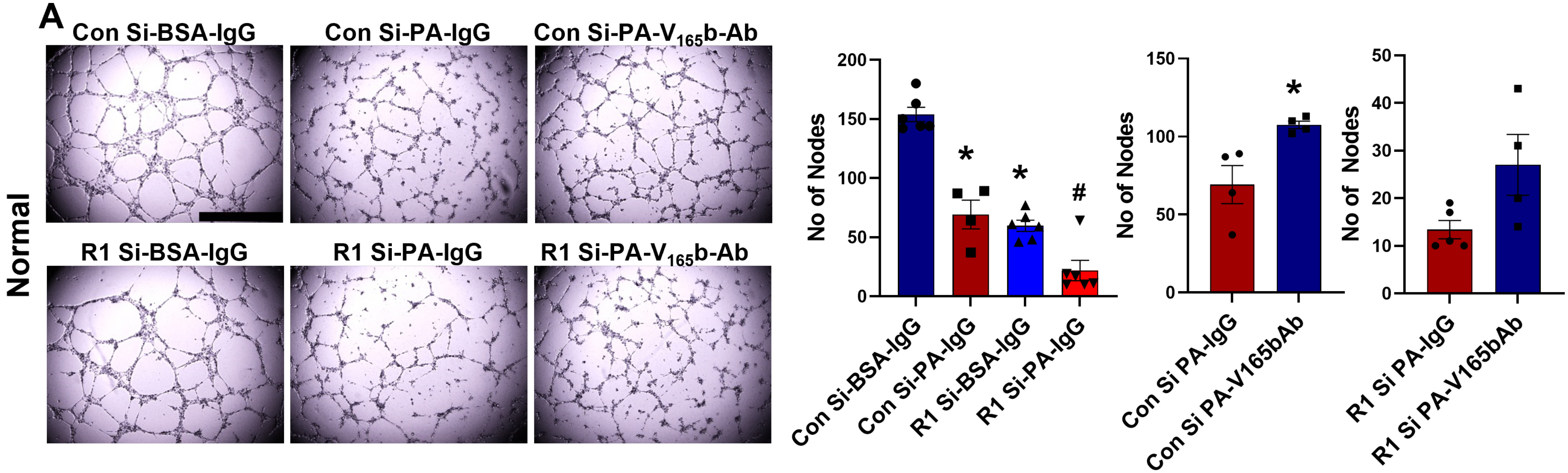

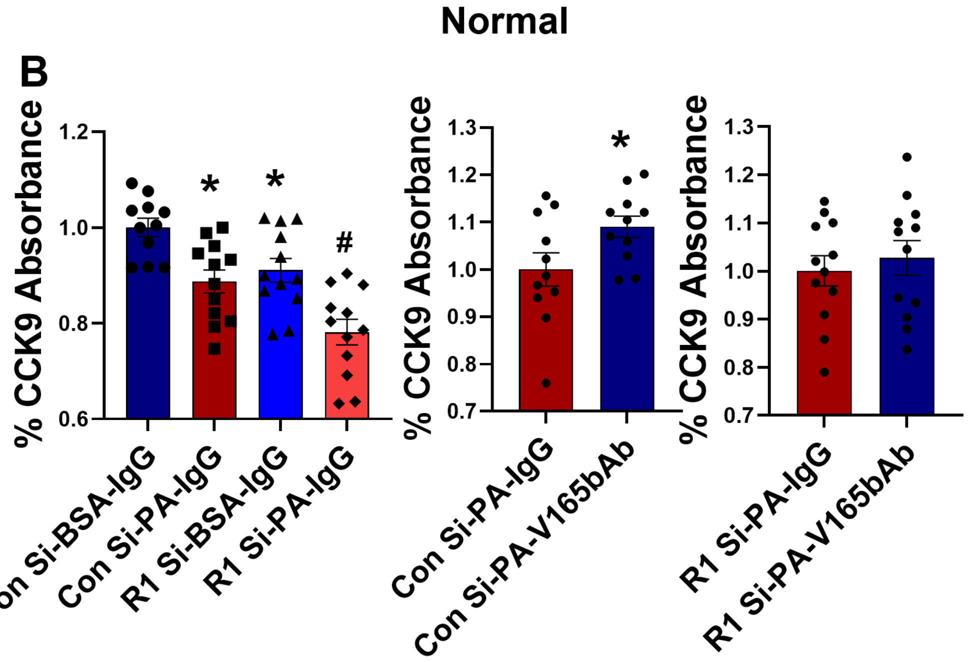

Figure S13. VEGFR1 silencing blocked the ability of VEGF165b-Ab to enhance the angiogenic capacity of endothelial cells in the in vitro T2D-model: A) In vitro tube formation assay of HUVECs transfected with non-targeting control siRNA (Con Si) or VEGFR1 siRNA (R1 Si), treated with BSA or BSA-Palmitic acid (500uM) and IgG or V165b-Ab (10ug/ml) for 24h under normal conditions. n≥4, One way ANOVA with Bonferroni select pair comparison. * indicates significantly different from Con Si-BSA-IgG, # indicates significantly different from Con Si-PA-IgG and R1 Si-PA-IgG. Scale-100um. B) Cell proliferation/survival assay of HUVECs transfected with non-targeting control siRNA (Con Si) or VEGFR1 siRNA (R1 Si), treated with BSA or BSA-Palmitic acid and IgG or V165b-Ab for 24h under normal growth conditions, n≥11, One way ANOVA with Bonferroni select pair comparison * indicates significantly different from Con Si-BSA-IgG, # indicates significantly different from Con Si-PA-IgG and R1 Si-PA-IgG. Unpaired T-test for IgG vs. V165b-Ab comparisons. *P<0.05 considered significant. Data Mean±SEM

Figure S14

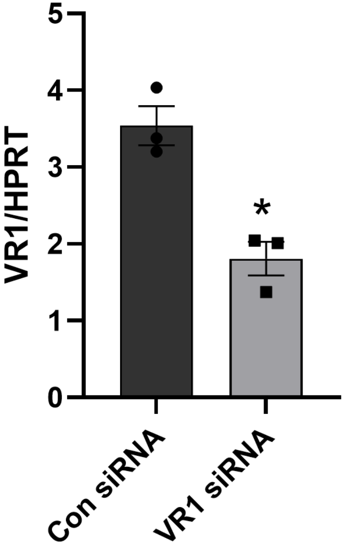

<u>Figure S14</u>. qPCR confirming VEGFR1 silencing in HUVECs transfected with VEGFR1 siRNA: qPCR analysis of VEGFR1 expression in non-targeting siRNA or VEGFR1 siRNA treated HUVECs. n=3, Unpaired T-test. *P<0.05 considered significant. Data Mean±SEM

Figure S15

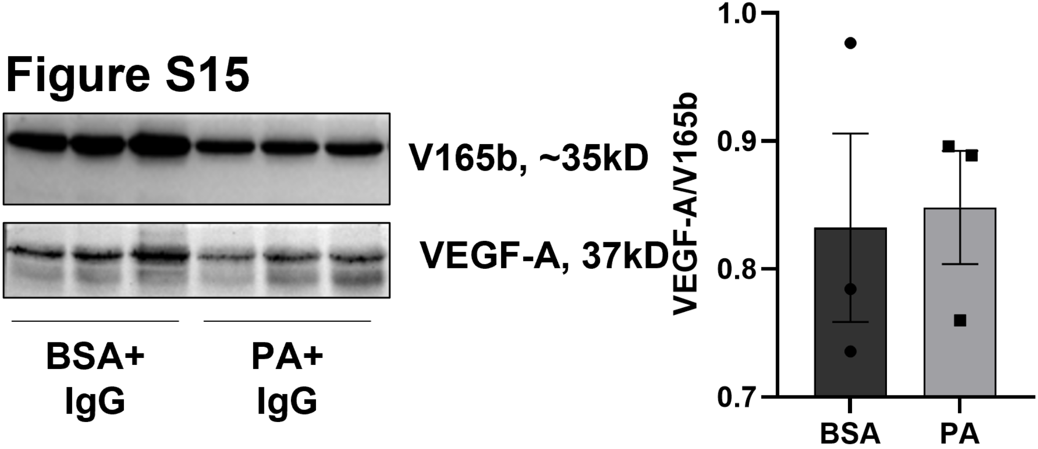

Figure S15. Palmitic acid did not induce significant differences in $VEGF_{165}b$ vs. VEGF-A levels in HSS HUVECs: Western blot analysis of $VEGF_{165}b$ and VEGF-A in HUVECs treated with BSA or PA under HSS conditions. n=3, Unpaired T-test. $^{*}P<0.05$ considered significant. Data Mean±SEM

Figure S16

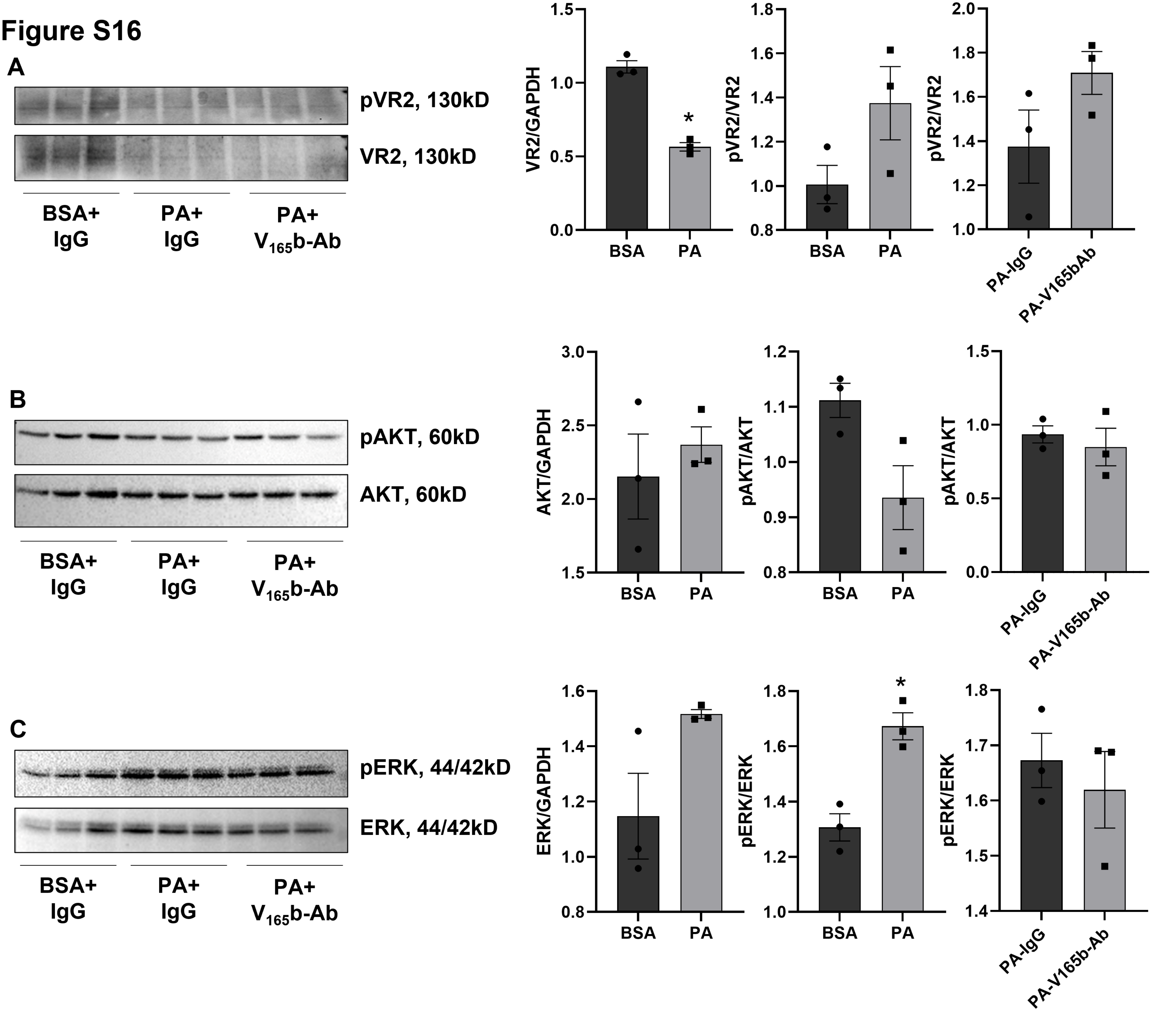

Figure S16. VEGF165b-inhibition does not induce VEGFR2-signaling in in vitro diabetic-PAD model: Western blot analysis of A) pVR2/VR2, B) pAKT/AKT, C) pERK/ERK in HSS-HUVECs treated with BSA+IgG, PA+IgG, PA+V165b-Ab. n=3, Unpaired T-test. *P<0.05 considered significant. Data Mean±SEM

Full westerns in Fig-1

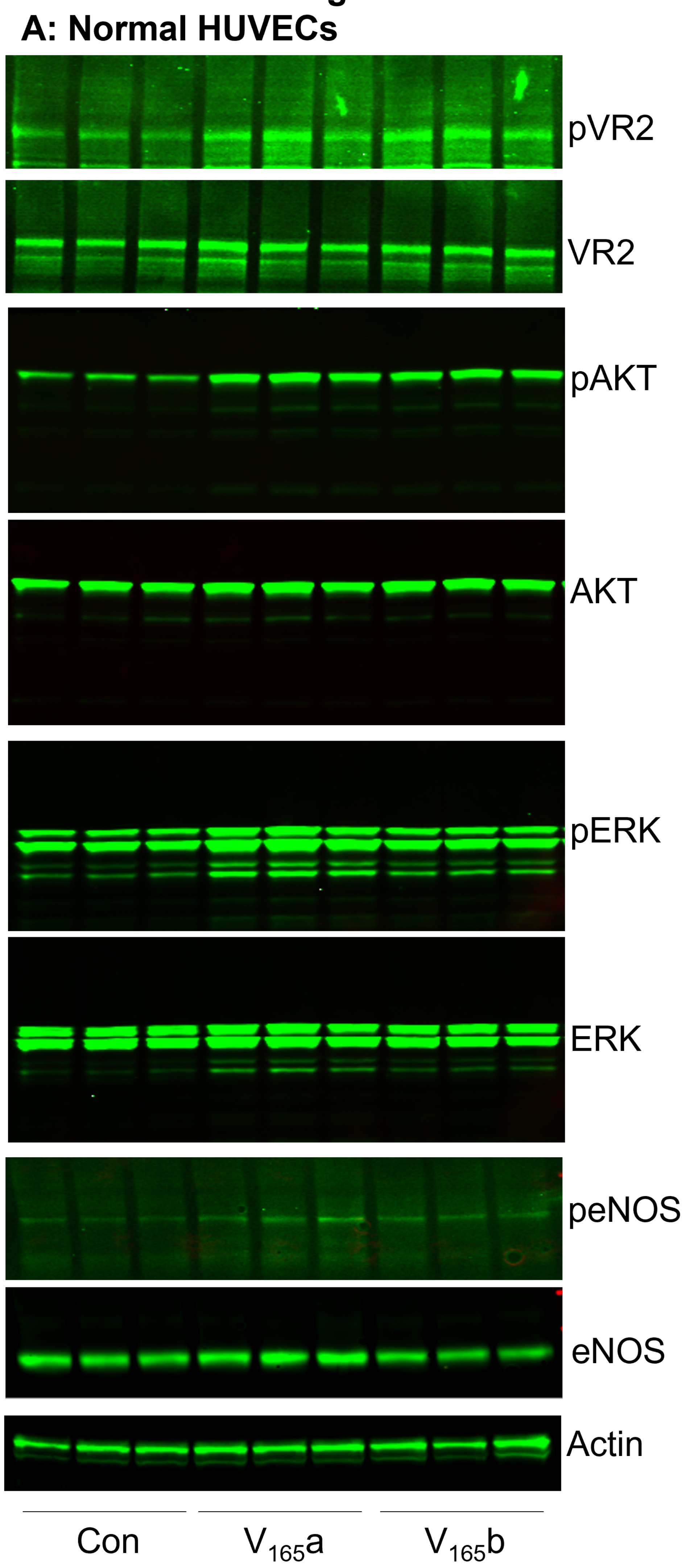

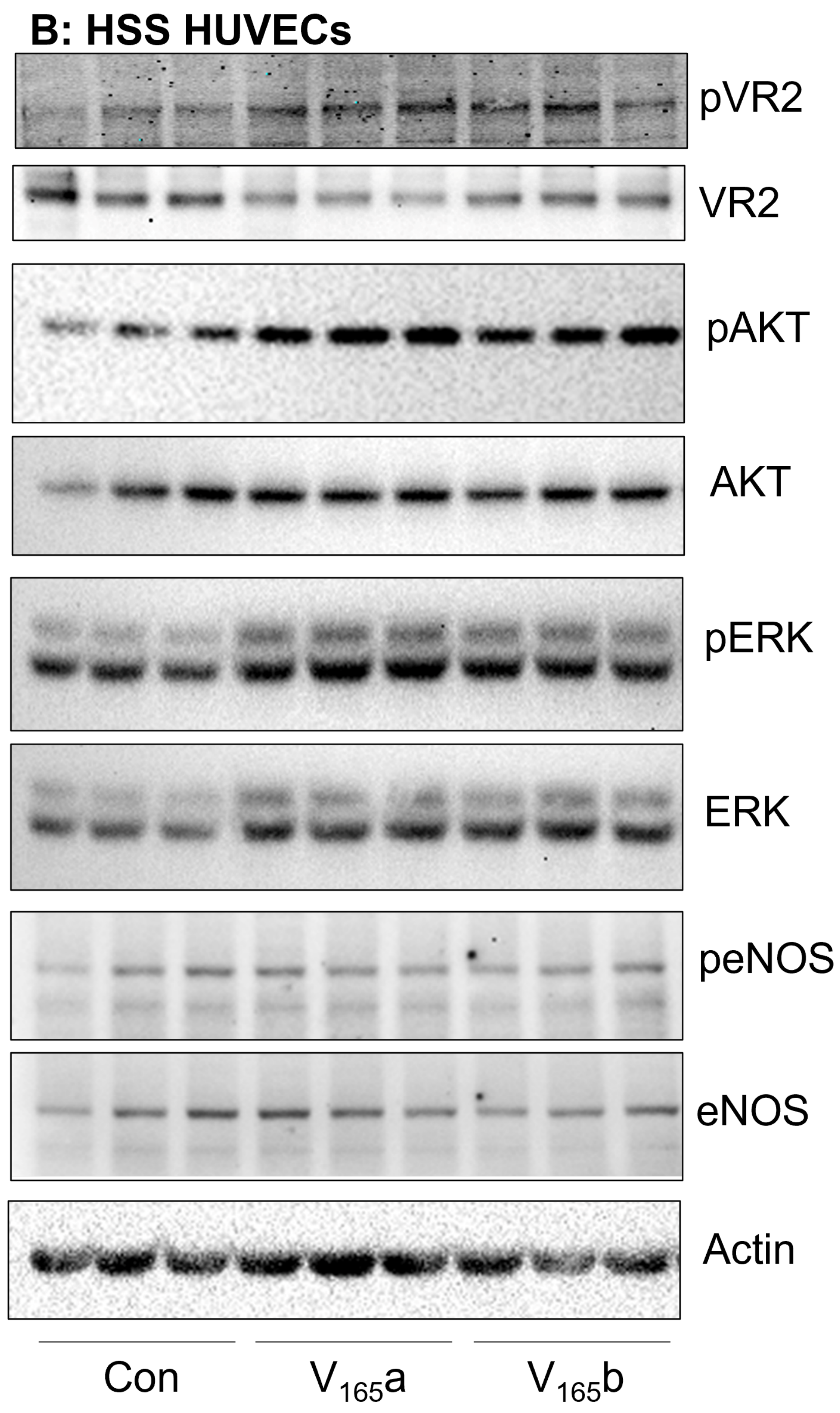

Full westerns in Fig-2

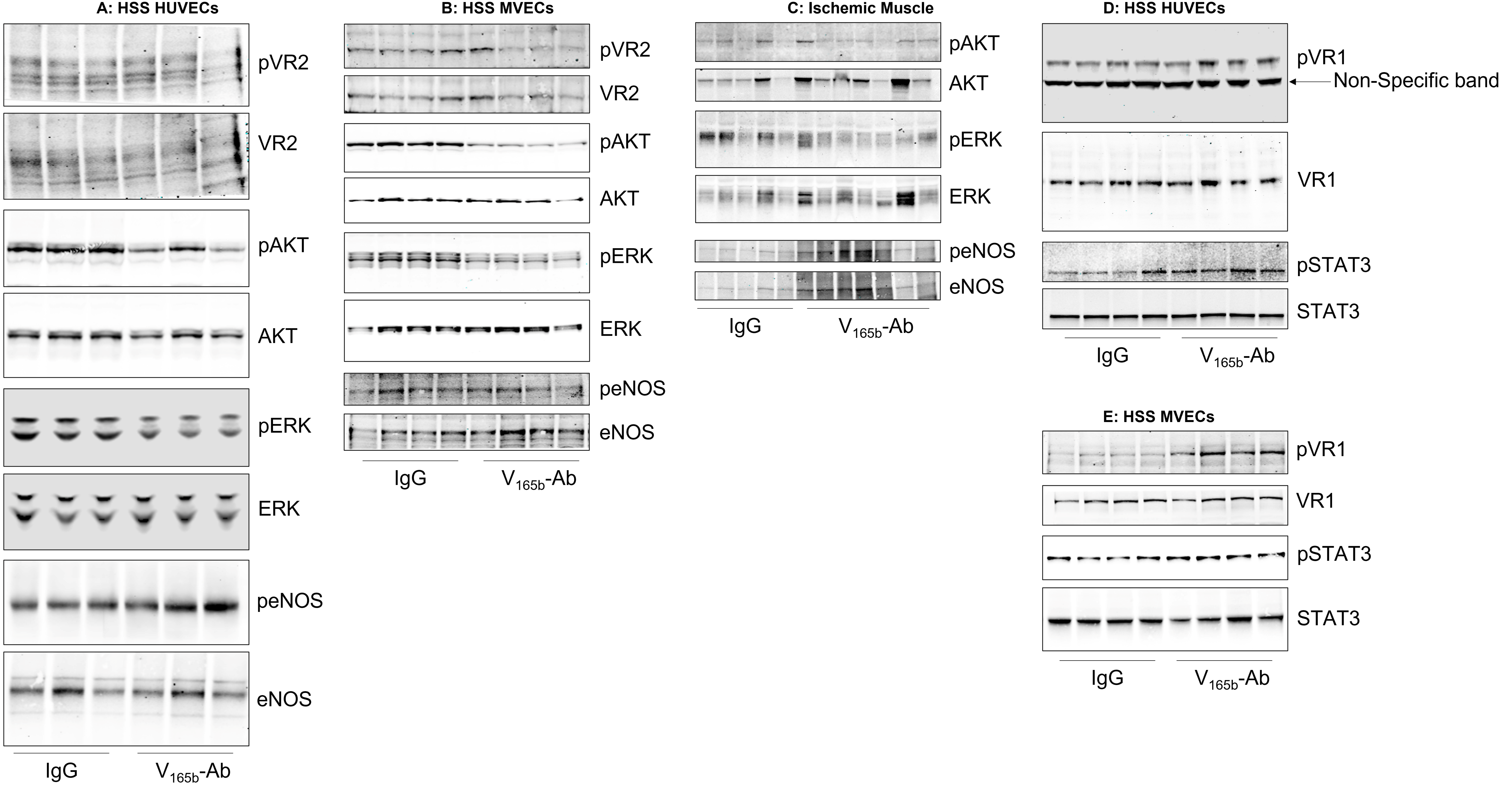

Full westerns in Fig-3

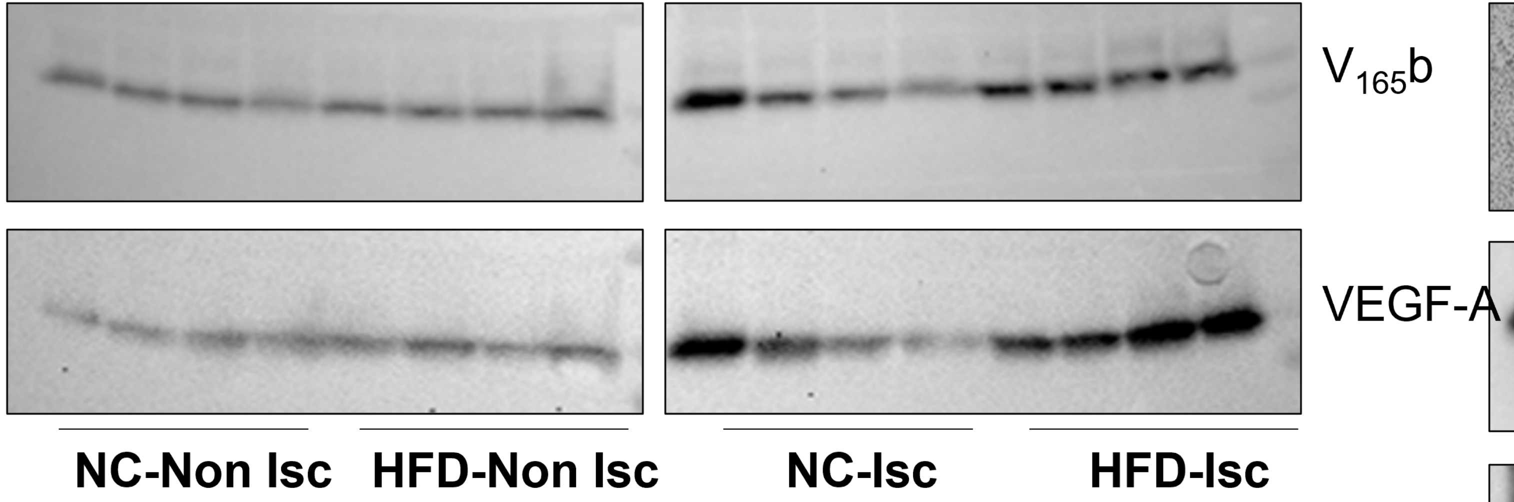

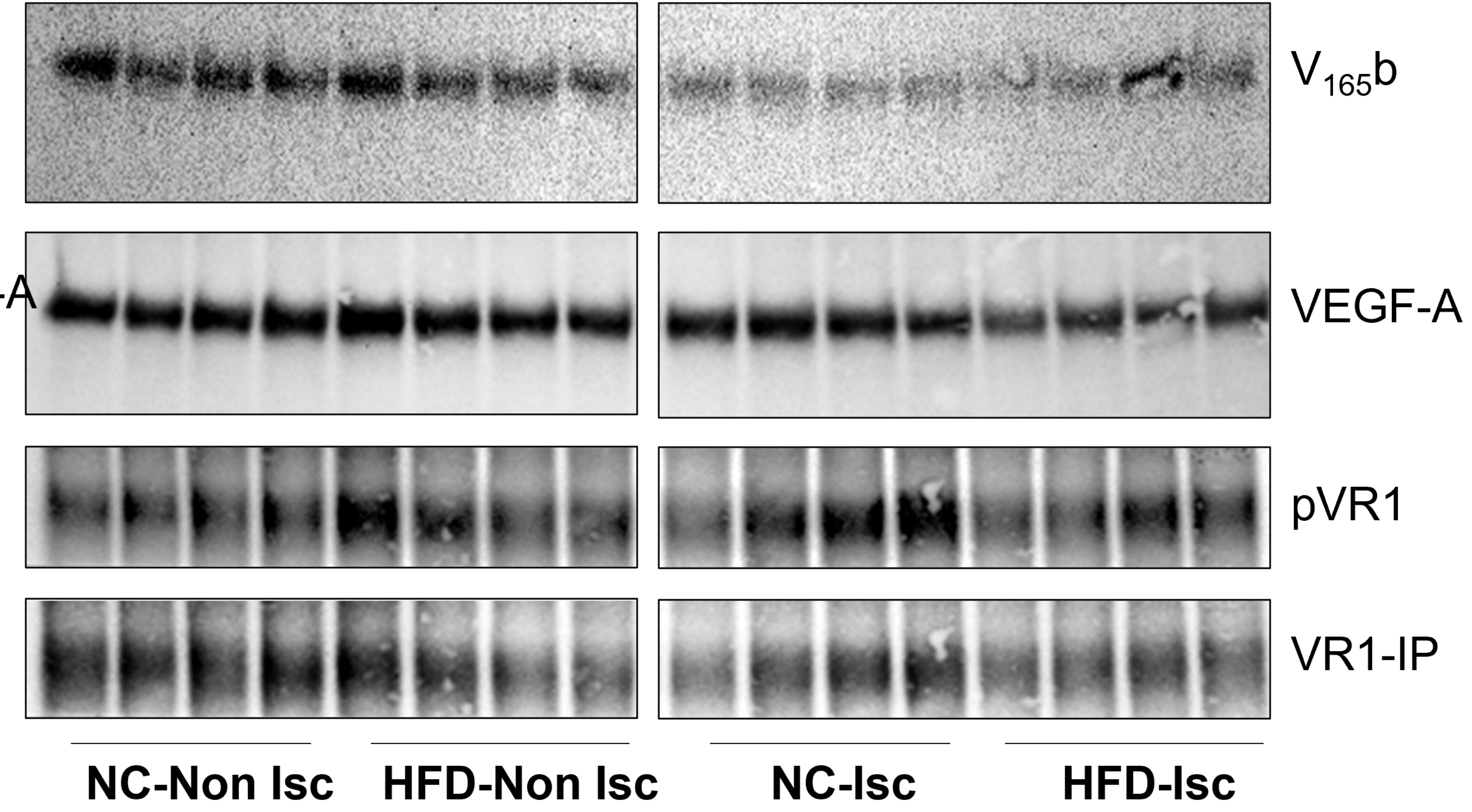

Full westerns in Fig-4

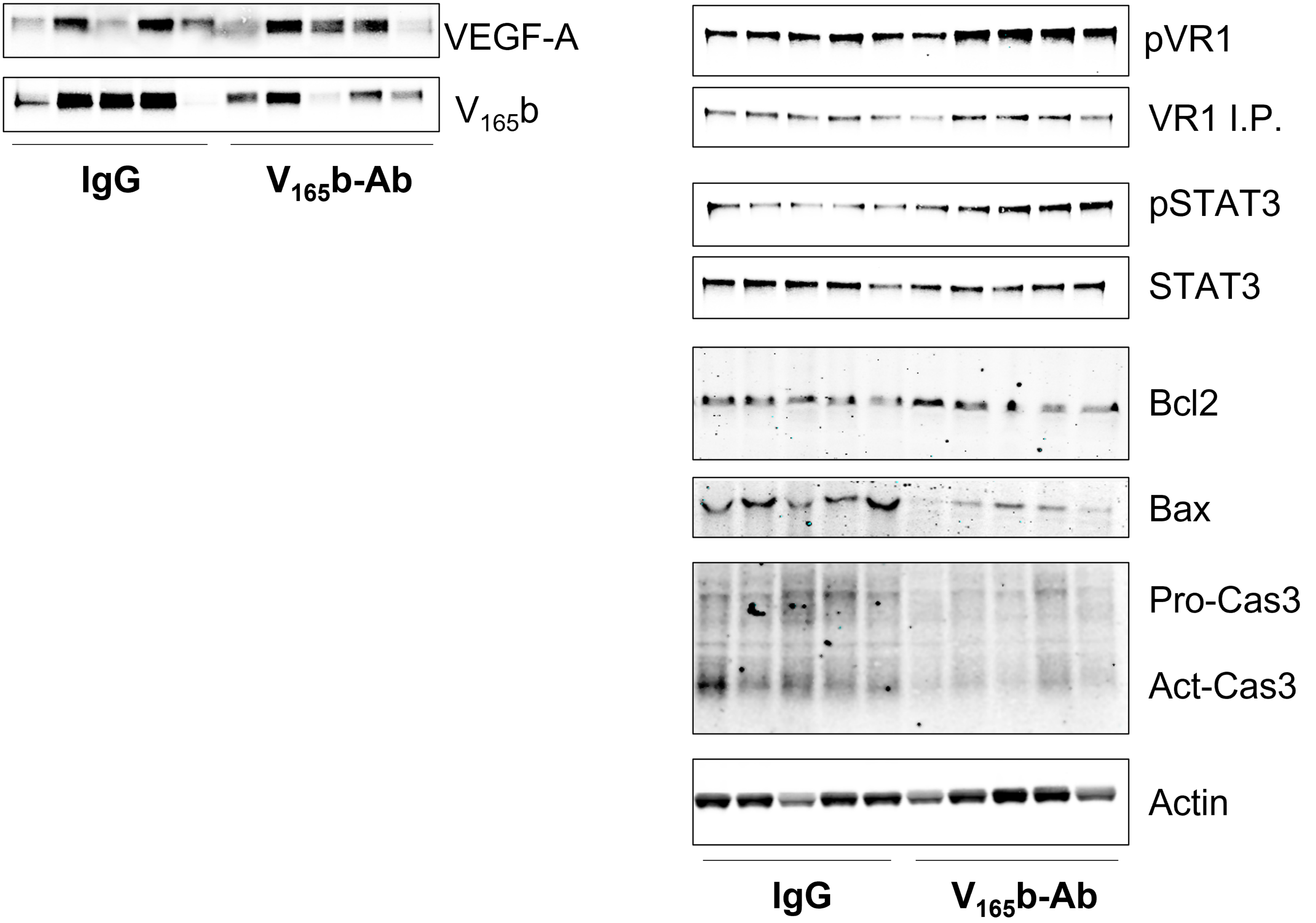

Full westerns in Fig-5

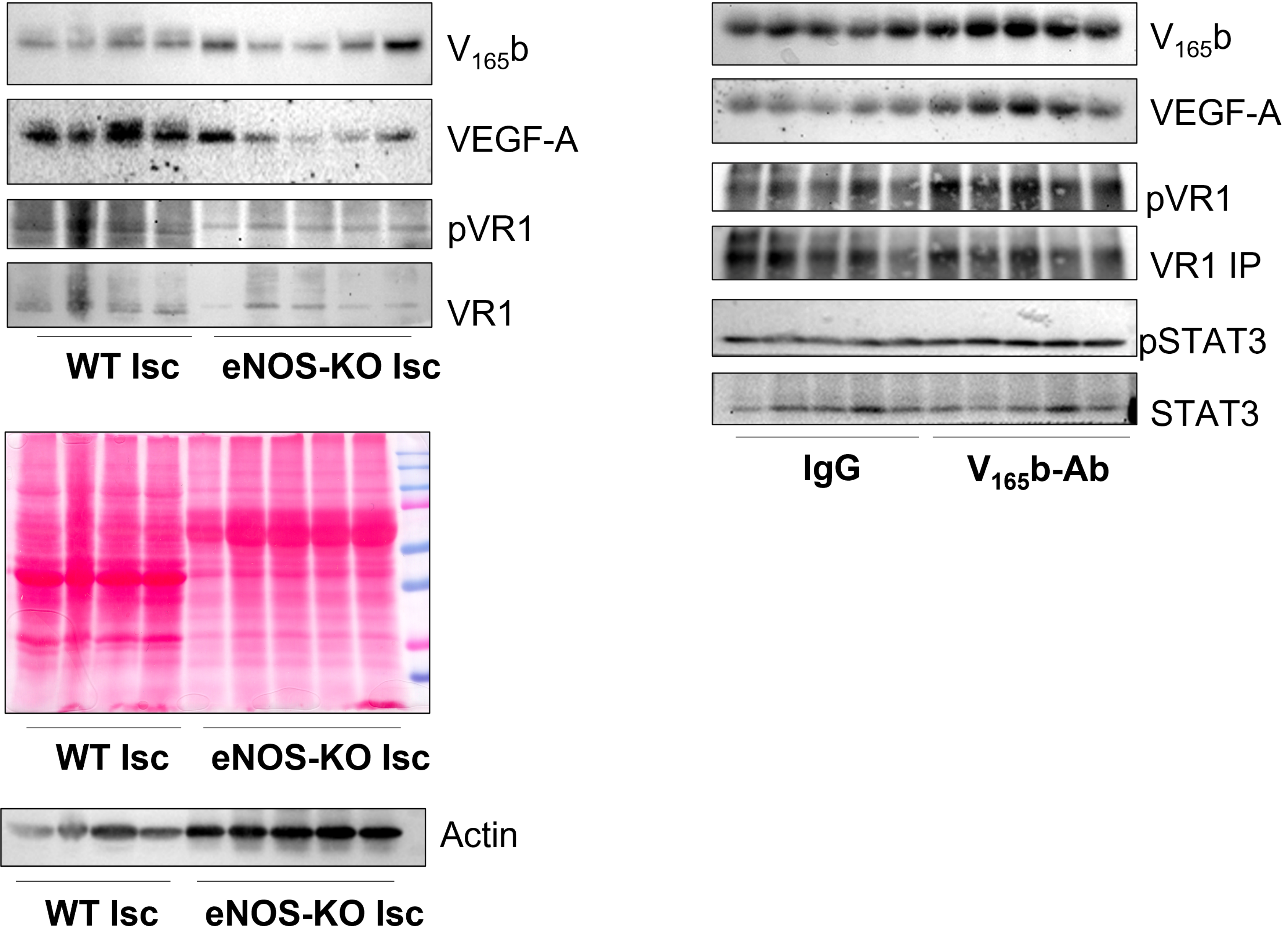

Full westerns in Fig-6

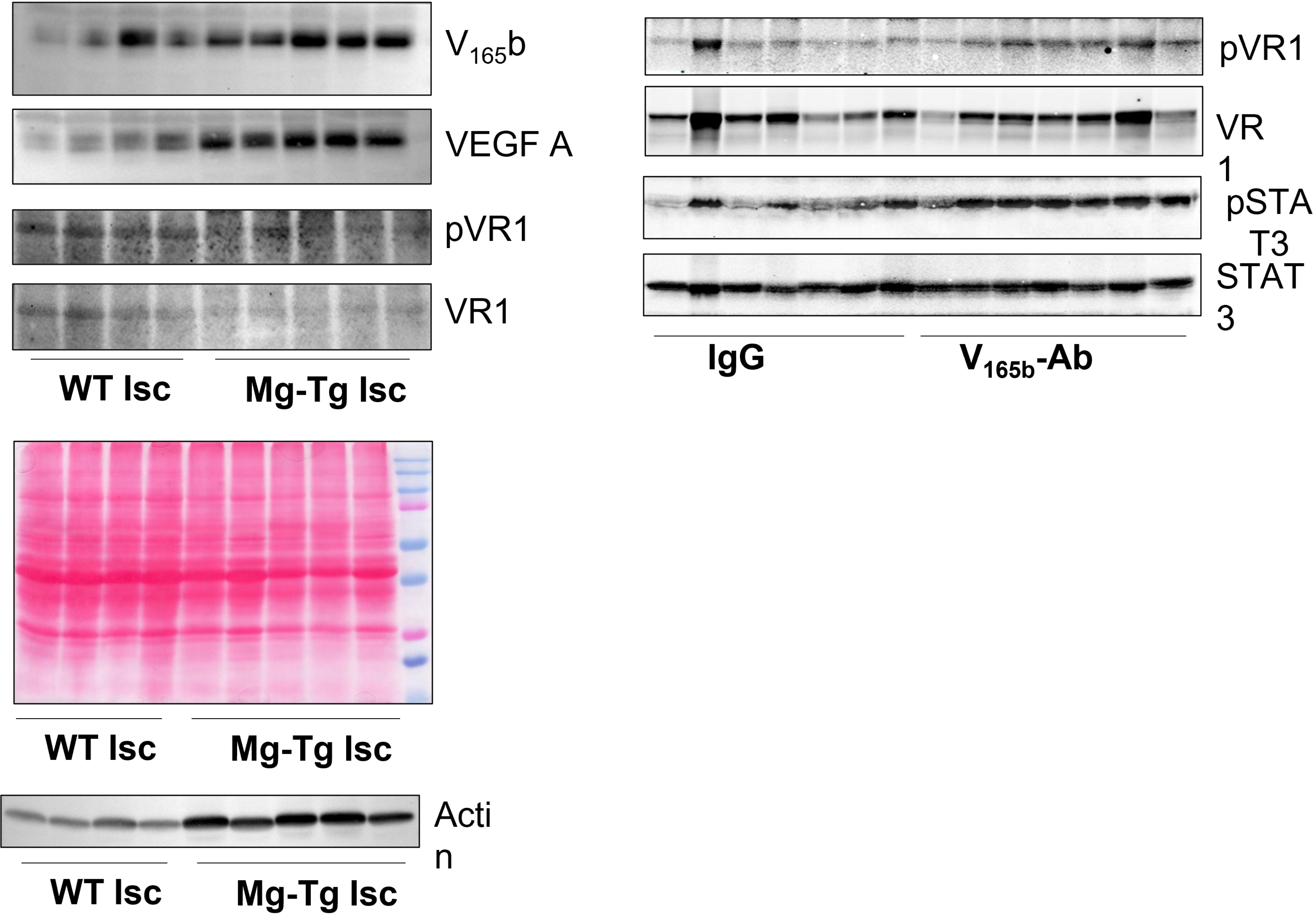

Full westerns in Supplement Fig-3: T2D-HLI

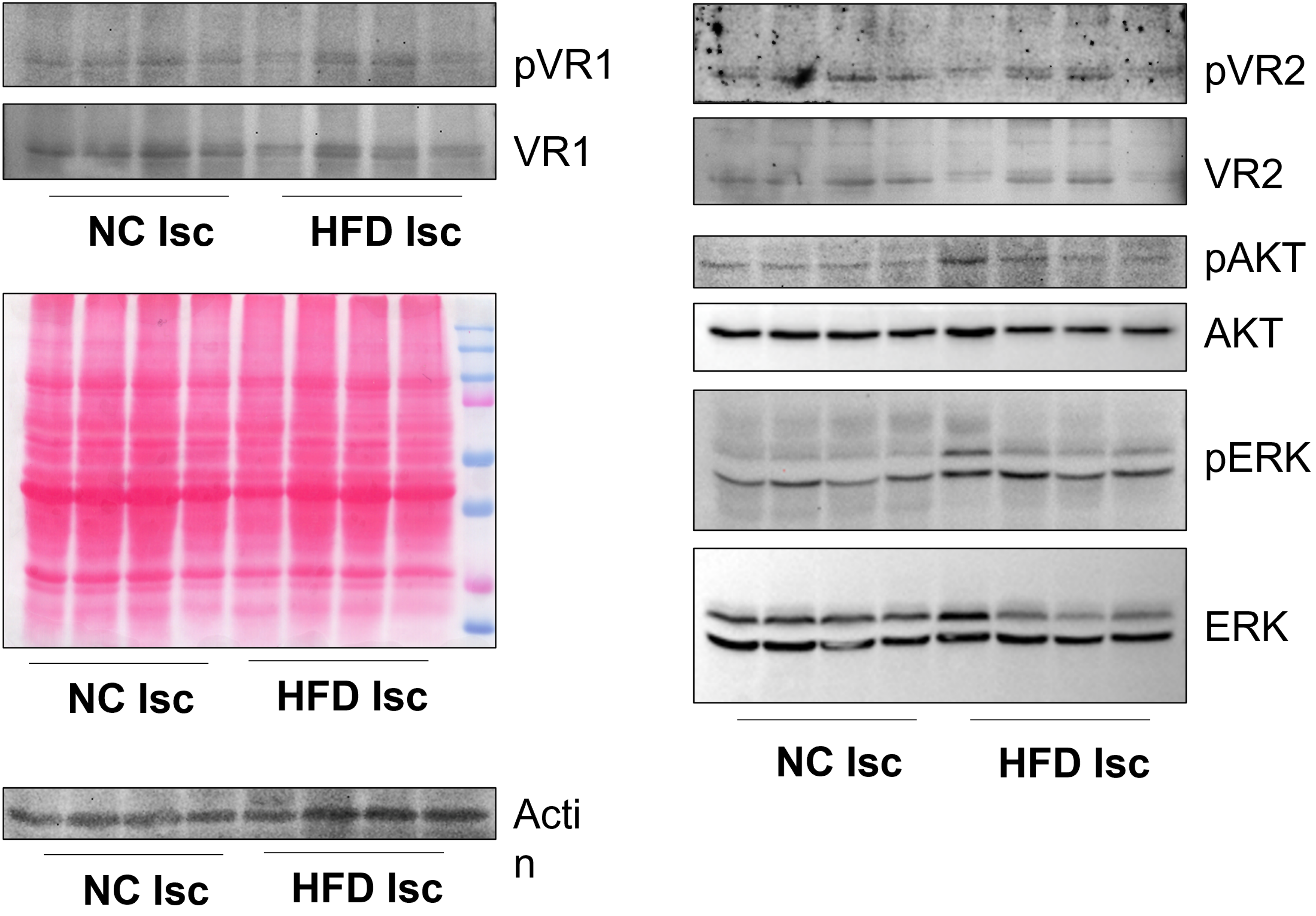

Full westerns in Supplement Fig-4: T2D-HLI

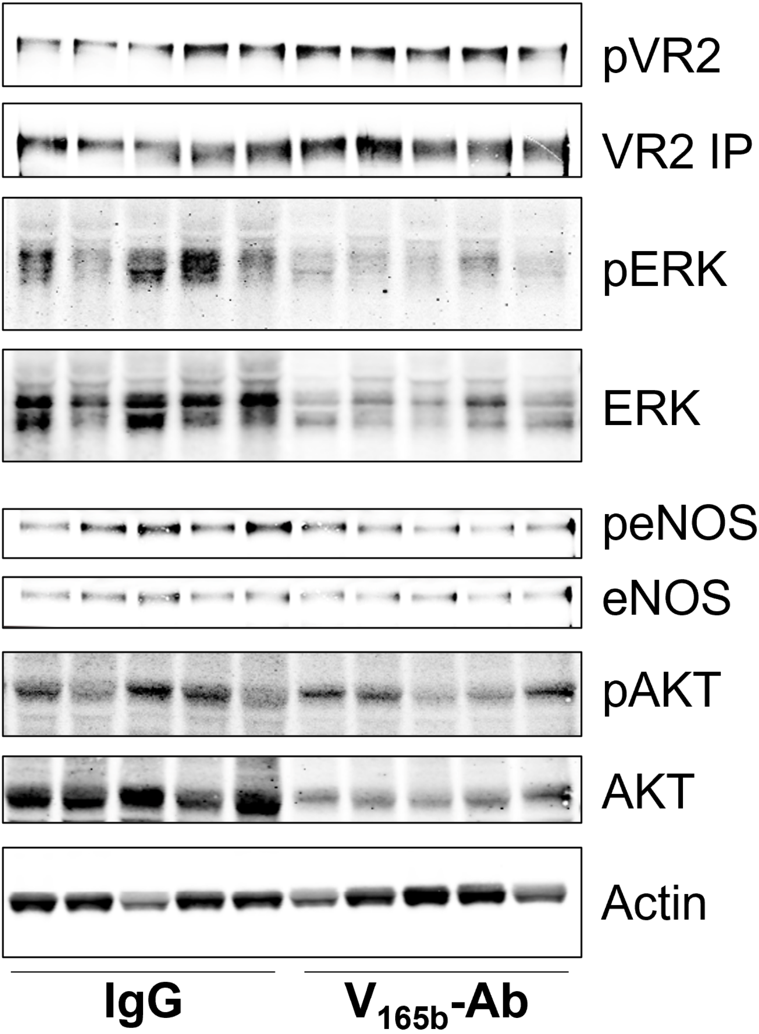

Full westerns in Supplement Fig-6: eNOS-KO-HLI

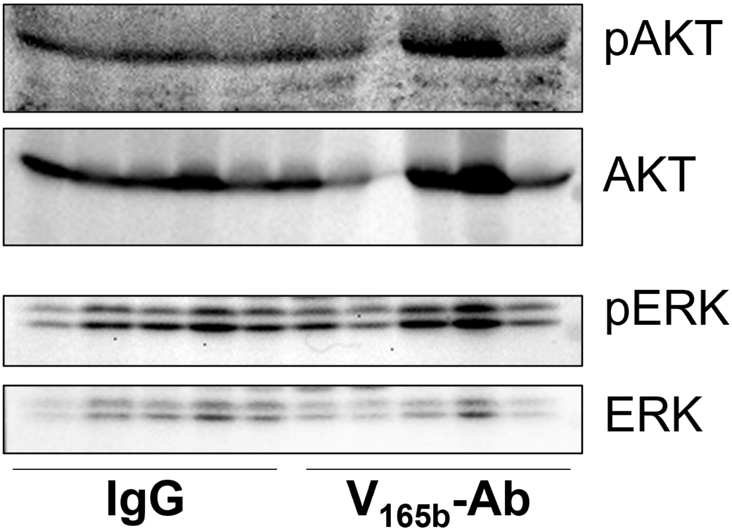

Full westerns in Supplement Fig-7: Mg-Tg-HLI

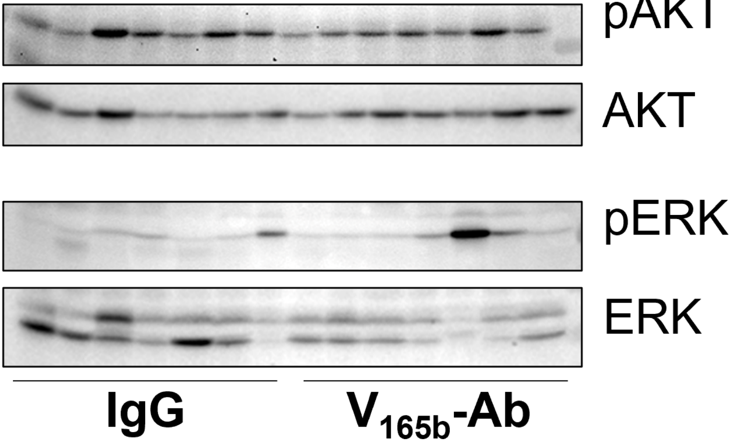

Supplement: Supplementary file 1 [file cells-11-02676-s001.zip › cells-1855565-SI.pdf]
